# New insights into binocular rivalry from the reconstruction of evolving percepts using model network dynamics

**DOI:** 10.3389/fncom.2023.1137015

**Published:** 2023-03-24

**Authors:** Kenneth Barkdoll, Yuhua Lu, Victor J. Barranca

**Affiliations:** Department of Mathematics and Statistics, Swarthmore College, Swarthmore, PA, United States

**Keywords:** neuronal networks, binocular rivalry, stimulus encoding, compressive sensing, non-linear dynamics, input-output mapping, autism

## Abstract

When the two eyes are presented with highly distinct stimuli, the resulting visual percept generally switches every few seconds between the two monocular images in an irregular fashion, giving rise to a phenomenon known as binocular rivalry. While a host of theoretical studies have explored potential mechanisms for binocular rivalry in the context of evoked model dynamics in response to simple stimuli, here we investigate binocular rivalry directly through complex stimulus reconstructions based on the activity of a two-layer neuronal network model with competing downstream pools driven by disparate monocular stimuli composed of image pixels. To estimate the dynamic percept, we derive a linear input-output mapping rooted in the non-linear network dynamics and iteratively apply compressive sensing techniques for signal recovery. Utilizing a dominance metric, we are able to identify when percept alternations occur and use data collected during each dominance period to generate a sequence of percept reconstructions. We show that despite the approximate nature of the input-output mapping and the significant reduction in neurons downstream relative to stimulus pixels, the dominant monocular image is well-encoded in the network dynamics and improvements are garnered when realistic spatial receptive field structure is incorporated into the feedforward connectivity. Our model demonstrates gamma-distributed dominance durations and well obeys Levelt's four laws for how dominance durations change with stimulus strength, agreeing with key recurring experimental observations often used to benchmark rivalry models. In light of evidence that individuals with autism exhibit relatively slow percept switching in binocular rivalry, we corroborate the ubiquitous hypothesis that autism manifests from reduced inhibition in the brain by systematically probing our model alternation rate across choices of inhibition strength. We exhibit sufficient conditions for producing binocular rivalry in the context of natural scene stimuli, opening a clearer window into the dynamic brain computations that vary with the generated percept and a potential path toward further understanding neurological disorders.

## 1. Introduction

A rich history of experiments demonstrate that when dissimilar constant visual stimuli are presented to each eye, humans typically perceive one of the monocular stimuli for a small period of time and then the other, with stochastic transitions between the two percepts as time advances (Dutour, 1760; Wheatstone, 1838). This phenomenon, referred to as *binocular rivalry*, has garnered numerous studies exploring its intricacies and emergence, from both psychophysics and neuroscience perspectives (Breese, 1909; Blake and Fox, 1974; Leopold and Logothetis, 1996; Polonsky et al., 2000; Tong and Engel, 2001; Wilke et al., 2003). A diverse range of explanations have been hypothesized (Levelt, 1965; Tong, 2001; Blake and Logothetis, 2002), with various mathematical models probing the efficacy of different proposed mechanisms (Matsuoka, 1984; Dayan, 1998; Wilson, 2003; Said and Heeger, 2013). Collectively, these studies have primarily underlined internally-generated noise, a slow adaptation mechanism, and cross-column inhibition as potentially pivotal, though a completely parsimonious framework for the irregular percept switching and underlying stimulus encoding is still an active interdisciplinary area of inquiry (Kovacs et al., 1996; Logothetis et al., 1996; Tong et al., 2006; Sterzer et al., 2009).

Since the percept evolves while the monocular inputs remain constant, binocular rivalry offers unique insight into the structure-function relationship in the visual system. Binocular rivalry also provides an informative vantage point for studying internal computations in the brain, and it has been broadly applied in studying consciousness as well as cognition (Wilson, 2013; Koch et al., 2016; Vattikuti et al., 2016). Perceptual alternations are well-documented across many non-human mammals (Sengpiel et al., 1994; Carter et al., 2020) and outside the domain of vision as well, including in olfaction (Zhou and Chen, 2009), audition (Hupe et al., 2008), and touch (Holcombe and Seizova-Cajic, 2008), suggesting perceptual multistability results from relatively universal organizing principles.

Theoretical investigations thus far have often well-replicated key experimental observations, but they have either utilized idealized rate models (Shpiro et al., 2009; Li et al., 2017) or relatively simple stimuli (Laing and Chow, 2002; Cohen et al., 2019; Wang et al., 2020), such as those distinguished by one parameter, akin to the orientation of a grating. These phenomenological models largely do not seek to estimate the stimulus information encoded by the network dynamics, rather focusing instead on winner-take-all behavior and multistability properties to probe the neural substrates of perceptual inference in binocular rivalry (Gershman et al., 2012). Considering that sequentially alternating percepts are relatively rarely encountered outside of laboratory settings yet robustly occur for disparate complex stimuli in experiments (Baker and Graf, 2009; Miller, 2013), with dominance characteristics often modulated by higher order image features (Alais and Melcher, 2007), it is of significant interest to address the retention of complex monocular stimuli in the network dynamics and the possible role of network structure in rivalry as well as potential information loss.

In this work, we provide a new verifiable framework for characterizing the interplay between visual system connectivity, non-linear network dynamics, and stimulus encoding in binocular rivalry, utilizing a multi-layer neuronal network model with two competing ocular dominance columns driven by disparate realistic monocular image inputs. This study is among the first to explicitly use stimulus reconstructions based on spiking neuronal network activity to investigate binocular rivalry and provides a direct method of testing theories regarding its neural substrates. Our two-layer mechanistic network model reflects the effective connectivity between the retina and V1 (Barlow, 1981; Spear et al., 1996). In the second layer, we introduce two pools of neurons with individually balanced dynamics driven by different images, cross-column competition *via* long-range connections from excitatory to inhibitory neurons, and spike-frequency adaptation to account for the common mechanisms believed to together be necessary for binocular rivalry. Each of these key assumptions is addressed in turn in this introduction.

We assume that the two downstream pools compete for dominance and each corresponds to a different attractor, such that when the network-averaged firing rate of one pool is significantly larger than that of the other pool, the percept corresponding to the more active pool is selected. Systems demonstrating competition among clusters of nodes display useful computational characteristics beyond decision making, such as sequence learning (McKinstry et al., 2016), classification (Krizhevsky et al., 2017), and signal restoration (Rutishauser et al., 2011). Competition in our model network stems from mutual inhibition between the pools that is facilitated by long-range connections from excitatory neurons in one pool to inhibitory neurons in the other, agreeing with experimental evidence that such long-range connections likely originate from excitatory neurons (Stettler et al., 2002; Douglas and Martin, 2004; Tamamaki and Tomioka, 2010; Binas et al., 2014).

Without slow adaptation or noise, a single percept may be selected for all time and the system would thus display winner-take-all behavior. In phenomenological models of binocular rivalry, adaptation is commonly implemented by either synaptic depression, in which neurotransmitters or vesicles are depleted over time, or spike-frequency adaptation (Freeman, 2005; Kilpatrick and Bressloff, 2010). There are two main slow currents that promote spike-frequency adaptation through gradual negative feedback, namely the non-inactivating muscarinic potassium current and the after-hyperpolarization (AHP) current, which together largely determine the firing threshold and slope of the neuronal voltage trace (Yamada et al., 1989; Ermentrout et al., 2001). Since the two main adaptation mechanisms both successfully decrease the excitability of the dominant pool after continued firing activity to help facilitate a switch in percept and there is no clear consensus regarding which mechanism is most closely implicated in binocular rivalry, we incorporate spike-frequency adaptation into our model for concreteness.

To account for the irregular nature of the percept switching in binocular rivalry, we assume each pool in the second layer demonstrates balanced dynamics in isolation (van Vreeswijk and Sompolinsky, 1996; Troyer and Miller, 1997), such that fluctuations in activity are internally generated. While here we assume irregular dynamics are the result of a chaotic attractor, noise is often instead explicitly incorporated into models of binocular rivalry and serves an analogous role in breaking symmetry to facilitate a new percept. Though either mechanism can provide an effective contribution to the stochastic switching, our choice of balanced dynamics reflects compelling experimental evidence that strong excitatory and inhibitory inputs into a given neuron are dynamically counteracted in time such that input fluctuations result in irregular spiking activity (Haider et al., 2006; Miura et al., 2007; London et al., 2010). Such balanced dynamics are theorized to have diverse functional benefits, facilitating robust spatial working memory (Lim and Goldman, 2014), effective predictive coding (Boerlin et al., 2013), efficient food source selection for honeybees (Borofsky et al., 2020), and successful mammalian learning (Fiete et al., 2007; Ingrosso and Abbott, 2019).

While our model exhibits highly non-linear and irregular dynamics with complex network structure, we nonetheless use coarse-graining techniques to derive an approximate linear input-output mapping. Linking the pixels composing the image stimuli to the evoked network dynamics, this map furnishes the reconstruction of the evolving dominant monocular image inputs and demonstrates the successful retention of stimulus information during binocular rivalry. We utilize a dominance metric based on the firing rates of the two downstream pools to identify the dominant pool during each period of dominance, exhibiting persistent bistability and irregular switching in the model dynamics. Using only the time-averaged activity data collected during a physiologically realistic dominance period, we are able to generate accurate dynamic percept reconstructions for both simple gratings as well as natural scene monocular inputs. The efficacy of these reconstructions is further improved when more biologically realistic spatial receptive fields are incorporated into the feedforward connectivity for the two pools, agreeing with the evolutionary selection of this network structure.

It is important to note that these reconstructions succeed despite our assumption that the monocular input images contain significantly more pixels than downstream neurons in the two pools, reflecting the compressive nature of the early visual system that is typically unaccounted for in model-based studies of binocular rivalry (Barlow, 1981; Barranca et al., 2014b). In the human visual system, for example, 150 million photoreceptors are processed downstream by only about 1.5 million retinal ganglion cells. Further downstream, information encoded by the millions of neurons in the lateral geniculate nucleus (LGN) is later expanded in the primary visual cortex (V1), which is composed of roughly 40 times as many neurons as the LGN (Spear et al., 1996). Compressive pathways are exhibited in most other sensory systems (Welker, 1976; Knudsen and Konishi, 1978; Mori et al., 1999; Brecht and Sakmann, 2002), and are hypothesized to play a central role in efficient coding in the brain (Barlow, 1961). Reflecting information loss through the sensory bottleneck and the initial time necessary for a monocular stimulus to dominate, our model demonstrates the experimentally documented transient period in the first few hundred milliseconds of binocular rivalry, resulting in heavily diminished percept reconstruction quality and likely reflecting the superposition of the monocular stimuli in the percept often reported during this period (Blake et al., 1991; Pearson and Brascamp, 2008).

By methodically varying the strengths of the monocular stimuli presented to the two pools, we establish agreement with Levelt's four laws, which commonly serve as a benchmark for theoretical investigations of perceptual bistability due to their ubiquity across years of experiments (Levelt, 1965). We additionally show that the dominance durations in our model are right-skewed and gamma distributed, agreeing with standard experimental observations (Kovacs et al., 1996). In exploring perturbations to the model dominance durations, we provide further insight into the precise mechanisms for the percept switching and how rivalry dynamics crucially depend on stimulus strength.

Finally, we utilize the flexible nature of our model network connectivity and our ability to directly probe its parameters to better understand neurological disorders. There is evidence that individuals with autism demonstrate a relatively slow rate of percept alternations in binocular rivalry (Robertson et al., 2013; Spiegel et al., 2019), and experiments also indicate that an imbalance in inhibitory and excitatory neuronal inputs may underlie autism spectrum disorders (Gao and Penzes, 2015; Nelson and Valakh, 2015; Rosenberg et al., 2015). Based on these two findings, we systematically scale down the strength of inhibition in our model, reflecting the potentially reduced inhibition in the autistic brain, and consequently produce increasingly long dominance durations. Providing new credence to the excitation/inhibition hypothesis for autism, this result strengthens the possibility of restoring balanced inhibition and excitation as a potential avenue for treating autism.

## 2. Results

### 2.1. Mathematical model of binocular rivalry with direct monocular image drive

**Figure 1:**
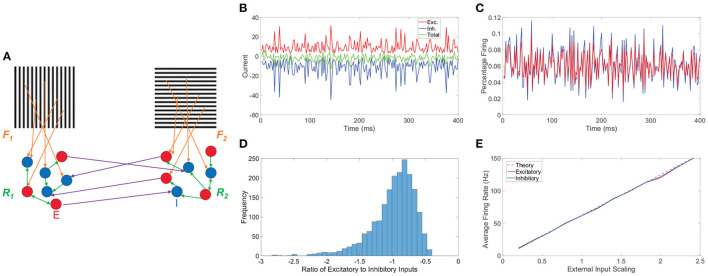
Network model and balanced dynamics. **(A)** Two-layer network with competing downstream pools schematic. Pool 1 and pool 2 are composed of integrate-and-fire neuronal networks driven by distinct monocular stimuli *via* feedforward connectivity matrices, **F_1_** and **F_2_**, respectively (orange). Recurrent interactions between neurons within pool 1 and pool 2 are given by matrices **R_1_** and **R_2_**, respectively (green). Long-range connections between neurons in the two pools (purple) originate at excitatory neurons (red, *E*) and terminate at inhibitory neurons (blue, *I*). **(B)** Time-evolving excitatory (red), inhibitory (blue), and total (green) inputs into a sample excitatory neuron in a balanced network. **(C)** The corresponding percentage of excitatory neurons firing (red) and inhibitory neurons firing (blue) as a function of time. **(D)** Histogram of the ratio between total excitatory input and total inhibitory input across neurons in the balanced network. **(E)** Gain curves depicting the population-averaged firing rates across a pool of downstream neurons as a function of the external input strength scaling. The excitatory population is plotted in red, the inhibitory population in blue, and the theoretical firing rate from Equation (8) in dashed purple. Note parameters are chosen such that theoretically the excitatory and inhibitory populations should have the same average firing rate. Panels **(B–E)** consider the balanced network dynamics of an isolated single downstream pool in the model prescribed by schematic **(A)** in the absence of both long-range connections between pools and spike-frequency adaptation. Parameters utilized are *R*_*EE*_ = *R*_*IE*_ = 1, *R*_*II*_ = −1.8, *R*_*EI*_ = −2, *N*_*E*_ = 1000, *N*_*I*_ = 1000, *f*_*E*_ = 1, *f*_*I*_ = 0.8, *m*_0_ = 0.5, *K* = 40, θ_*E*_ = 1, and θ_*I*_ = 0.8.

The two-layer network model we consider has two competing pools of downstream neurons that are each driven by distinct monocular image inputs, as represented by the schematic in Figure 1A. In light of several lines of experimental evidence indicating a link between percepts reported in binocular rivalry and the activity in both V1 (Logothetis et al., 1996; Polonsky et al., 2000) and other parts of the visual system (Haynes et al., 2005), we view the feedforward architecture of our model as a phenomenological reflection of the effective connectivity between photoreceptor output in the retina and V1, abstracting over the detailed connectivity structure and diverse neuron types in between these areas (Field and Chichilnisky, 2007; Anderson et al., 2011). Since it may potentially be the case that a hierarchy of several network layers are all partially responsible for various features of binocular rivalry (Wilson, 2003), we aim to include more general characteristics in our network connectivity and focus on dynamic percept encoding in the context of the key mechanistic contributing factors.

Each downstream pool is composed of *N* neurons, such that *N*_*E*_ are excitatory and *N*_*I*_ are inhibitory, with each neuron exhibiting pulse-coupled integrate-and-fire (I&F) dynamics. Though relatively idealized, the I&F model has been shown to well replicate experimentally observed subthreshold voltage dynamics and has been utilized to great effect in numerous large-scale model-based studies (Abbott, 1999; Rauch et al., 2003; Burkitt, 2006; Rangan and Cai, 2006; Mather et al., 2009; Barranca et al., 2014a). In formulating the detailed recurrent network connectivity and resultant dynamical regime, we utilize a balanced network configuration within each pool (van Vreeswijk and Sompolinsky, 1996; Miura et al., 2007; Barranca and Zhou, 2019; Barranca et al., 2019), producing the asynchronous dynamics in the dominant pool that underlies the stochastic switching phenomenon and our derivation of the network input-output transformation. While there may be other plausible noise-based mechanisms for the variability observed in dominance durations (Freeman, 2005), balanced networks have recently been incorporated into the study of binocular rivalry (Cohen et al., 2019; Wang et al., 2020) and largely produce dynamics that agree with core experimental findings on both the distribution of dominance durations and the variability of neuronal activity more broadly.

The voltage (membrane-potential) of the *i*^th^ neuron in the *k*^th^ population of the first downstream pool in the network, ${v_{1}}_{k}^{i}$, has activity dictated by the dynamical system below (subscripts *k* = *E* and *k* = *I* denote excitatory and inhibitory neurons, respectively)


(1a) $$\begin{array}{ll} \frac{dv_{1}{}_{k}^{i}}{dt}= & -g_{L}(v_{1}{}_{k}^{i}-V^{Re})+\sum_{\begin{array}{c} j=1\\ j\neq i \end{array}}^{N_{E}}R_{1}{}_{kE}^{ij}\sum_{l}\delta (t-\tau_{1}{}_{E}^{jl})\;\\ & +\sum_{\begin{array}{c} j=1\\ j\neq i \end{array}}^{N_{I}}R_{1}{}_{kI}^{ij}\sum_{l}\delta (t-\tau_{1}{}_{I}^{jl})+\sum_{\begin{array}{c} j=1\\ j\neq i \end{array}}^{N_{E}}C_{2}{}_{kE}^{ij}\sum_{l}\delta (t-\tau_{2}{}_{E}^{jl})\\ & +\sum_{j}F_{1}{}_{k}^{ij}p_{1}{}^{j}, \end{array}$$

(1b) $$\begin{array}{l} \frac{d{\theta_{1}}_{k}^{i}}{dt}=-\lambda \left({\theta_{1}}_{k}^{i}-\theta_{k}\right), \end{array}$$

evolving from reset potential, *V*^*Re*^, until increasing enough to reach its firing threshold, ${\theta_{1}}_{k}^{i}$, at which time the neuron spikes. Once a neuron fires, its voltage is instantaneously reset to the value *V*^*Re*^ and the voltages of all post-connected neurons are adjusted upon integrating over the Dirac delta functions δ(·) in Equation (1a). The spike times for the *i*^th^ neuron in the *k*^th^ population of the first pool are denoted ${\tau_{1}}_{k}^{il}$ and are indexed by *l* = 1, 2, …  in ordering the firing events in time. Note that the voltage dynamics of the neurons in the second downstream pool are analogously defined except the locations of the initial subscripts 1 and 2 in Equation (1) are interchanged.

The *N*×*N* recurrent connectivity matrix for the first downstream pool, **R_1_**, determines the instantaneous neuronal interactions within the pool upon firing events and is indexed such that ${R_{1}}_{kl}^{ij}$ denotes the recurrent connection strength between the *i*^th^ post-connected neuron in the *k*^th^ population and the *j*^th^ pre-connected neuron in the *l*^th^ population. Each entry of the recurrent connectivity matrix is prescribed by a Bernoulli distribution, where ${R_{1}}_{kl}^{ij}=R_{kl}/\sqrt{K}$ with probability *K*/*N*_*l*_ and ${R_{1}}_{kl}^{ij}=0$ otherwise. Thus, on average, a given downstream neuron will be post-connected to *K* excitatory and *K* inhibitory neurons within its pool, where the excitatory connection strength *R*_*kE*_ is positive and the inhibitory connection strength *R*_*kI*_ is negative. The recurrent connectivity is assumed sparse in this model, as typically observed in experiment (He et al., 2007; Ganmor et al., 2011), implying 1≪*K*≪*N*_*E*_, *N*_*I*_.

Each downstream pool corresponds to a column of neurons with a specific ocular dominance preference, which is well-documented in V1 (Blasdel, 1992; Issa et al., 1999). Reflecting ocular preference, a neuron in a given downstream pool receives external drive determined by its corresponding monocular stimulus, which is mediated by a feedforward connectivity matrix. In the first pool, for example, the corresponding monocular stimulus is given by a constant image input vector **p_1_** with *n* = 10*N*_*E*_ total pixel components, modeling the large number of photoreceptors relative to downstream neurons in V1 (also relative to the number of neurons in earlier layers, such as the retinal ganglion cells or neurons in the LGN). We have verified that a less extreme compression ratio, focused on connectivity between alternative specific layers, may be used with minimal impact on our analysis. The feedforward connectivity matrix, **F_1_k**, determines the connections between the upstream and downstream layers, where ${F_{1}}_{k}^{ij}$ denotes the feedforward connection strength between the *j*^th^ component of the stimulus vector, ${p_{1}}^{j}$, and the *i*^th^ neuron in the *k*^th^ population of the first pool. Note, however, we do not consider neurons with orientation selectivity (Ferster et al., 1996; Tao et al., 2006) and we view the monocular image input as a single patch of the visual scene sampled by the downstream pool in V1 (Hubel and Wiesel, 1962) for analytical tractability and more direct mechanistic insights that apply to stimuli beyond simple oriented gratings. With the model network connectivity characterized, we now proceed to underline the three main model features that are together sufficient to produce perceptual alternations in binocular rivalry.

#### 2.1.1. Irregular firing activity

Irregular firing activity is internally generated by the recurrent interactions among the downstream neurons, as we assume the external input vectors forcing the two pools are constant, such that the expected external drive into a downstream neuron in the *k*^th^ population of either pool is $\sqrt{K}f_{k}m_{0}$. Here, *m*_0_ and *f*_*k*_ are O(1) parameters applying to both pools, where *m*_0_ scales the overall external drive strength for the two populations and *f*_*k*_ further scales the external drive strength for the *k*^th^ population only. For analytical tractability in our initial investigation, we assume the feedforward connectivity matrices are sparse with entries determined by Bernoulli distributions akin to the recurrent connectivity matrices. Later, in Section 2.5, we instead consider the impact of utilizing more realistic spatially localized feedforward connectivity.

#### 2.1.2. Competition

Competition between the two downstream pools is fostered by long-range connections from excitatory neurons in one pool to inhibitory neurons in the other pool. The long-range connections starting from neurons in the second pool are given by matrix **C_2_**, where ${C_{2}}_{kl}^{ij}$ denotes the long-range connection strength between the *i*^th^ post-connected neuron in the *k*^th^ population of the first pool and the *j*^th^ pre-connected neuron in the *l*^th^ population of the second pool. The entries of **C_2_** are prescribed similarly to those of the recurrent connectivity matrices except the only non-zero long-range connections possible are from excitatory to inhibitory neurons, so ${C_{2}}_{IE}^{ij}=R_{IE}/\sqrt{K}$ with probability *K*/*N*_*E*_ and ${C_{2}}_{kl}^{ij}=0$ otherwise. In this case, when one pool is highly active, it will send numerous excitatory inter-pool impulses to inhibitory neurons in the other pool and thereby tends to suppress the activity of the competing neurons. Though a host of mechanisms for inter-pool competition exists, such long-range excitatory connections are a plausible and biologically feasible apparatus for inter-pool competition (Stettler et al., 2002; Douglas and Martin, 2004; Tamamaki and Tomioka, 2010; Binas et al., 2014), and they have been included in prior realistic models of binocular rivalry (Wang et al., 2020).

In an isolated pool that receives no cross-pool connections, reminiscent of the dominant pool that receives only weak competitive drive from the suppressed pool, it is important to underline that if the recurrent connectivity strength parameters, *R*_*kl*_, are O(1), then only $O(\sqrt{K})$ excitatory impulses are needed for a constituent neuron to fire given an O(1) firing threshold. Hence, the mean excitatory and inhibitory inputs into each downstream neuron are each in total of the same order as the default firing threshold, reflecting the dynamic balance of large excitatory and inhibitory inputs observed in many experimental settings (Britten et al., 1993; Haider et al., 2006; Miura et al., 2007; London et al., 2010; Xue et al., 2014). As a result, intermittent fluctuations in neuronal input are largely responsible for firing events as well as their irregular distribution. Since the excitatory and inhibitory inputs into a given downstream neuron in the balanced state dynamically cancel over time, a nearly constant level of asynchronous activity is typically produced across neurons in the dominant pool (van Vreeswijk and Sompolinsky, 1996; Barranca et al., 2019).

#### 2.1.3. Spike-frequency adaptation

Spike-frequency adaptation is the final major feature incorporated into the model (Brown and Adams, 1980; Benda and Herz, 2003; Barranca et al., 2014a), supported by a large body of theoretical evidence that underlines the resultant decrease in neuronal firing rate over time in response to a constant stimulus as a core contributor to the percept switching in binocular rivalry. In our model, adaptation arises from a dynamic firing threshold for each neuron, rather than a static threshold as in traditional balanced networks. As time progresses, this dynamic firing threshold generally rises for the dominant pool and consequently increases the excitation needed for its neurons to undergo action potentials. Multiple subsequent spikes occurring close in time sum to produce an accumulated increase in firing threshold and ultimately reduce a neuron's firing frequency. In particular, the firing threshold of the *i*^th^ neuron in the *k*^th^ population of the first downstream pool, ${\theta_{1}}_{k}^{i}\left(t\right)$, increases by a fixed positive constant ϕ at the moment that neuron fires. Between firing events, ${\theta_{1}}_{k}^{i}\left(t\right)$ evolves according to Equation (1b), such that the dynamic firing threshold decays to the constant non-adapted firing threshold for all neurons in the *k*^th^ population, θ_*k*_, in the absence of firing events, with speed dictated by decay rate constant λ. The voltages and thresholds are non-dimensionalized such that *V*^*Re*^ = 0, θ_*E*_ = 1, and θ_*I*_ = 0.8, with $g_{L}=50{\text{s}}^{-1}$ corresponding to the standard membrane-potential time-scale of 20 ms (McLaughlin et al., 2000; Brette et al., 2007; Barranca et al., 2014b).

In the context of this study, adaptation further facilitates rivalry since it causes the activity of the dominant pool to weaken with time, giving the suppressed pool the opportunity to again dominate as the firing thresholds of its neurons decrease and consequently the overall firing activity of the suppressed pool increases with time. Without relatively irregular neuronal dynamics, such alternations in dominance could occur almost periodically with highly correlated dominance durations. In order to agree with the gamma-distributed dominance durations typical in experimental data (Kovacs et al., 1996), irregularity is intrinsically introduced through balanced network structure, and prior theoretical work suggests that binocular rivalry may only occur with the appropriate mix of irregularity and adaptation (Shpiro et al., 2009).

Before turning to the rivalrous behavior of the full model, we provide useful insight into the model network dynamics by underlining several main characteristics of balanced activity for a single isolated pool in the absence of spike-frequency adaptation in Figure 1. For a sample downstream neuron, we plot in Figure 1B its total excitatory and inhibitory inputs, which dynamically cancel over time and are much larger in magnitude than the static firing threshold, causing the neuronal voltage to only sporadically reach the firing threshold. As a result, on the population scale, we observe in Figure 1C a nearly constant level of asynchronous firing activity with only a relatively small percentage of neurons spiking at any point in time. For a given neuron, the time-averaged ratio between its total excitatory and total inhibitory input is primarily near −1. Since this quotient is always negative when well-defined and a ratio larger in magnitude than −1 implies an excess of excitatory input, excitatory to inhibitory input ratios near −1 demonstrate that on a neuron-by-neuron basis the excitatory and inhibitory inputs are largely proportional. A histogram of these ratios across the pool of neurons is given in Figure 1D, showing a clear center near −1 and thus widespread balance. Despite the highly non-linear voltage dynamics of neurons on an individual level, the network firing rate response properties of the isolated balanced network are quite linear. In Figure 1E, we adjust the external input by increasing the overall scaling strength *m*_0_ and depict the resultant population-averaged firing rates. We observe that as the mean external drive is increased, the population-averaged firing rate of both the excitatory and inhibitory populations linearly amplifies for this finite network realization and agrees with the theoretical gain curve derived in the large-network limit in Section 4.1.

**Figure 2:**
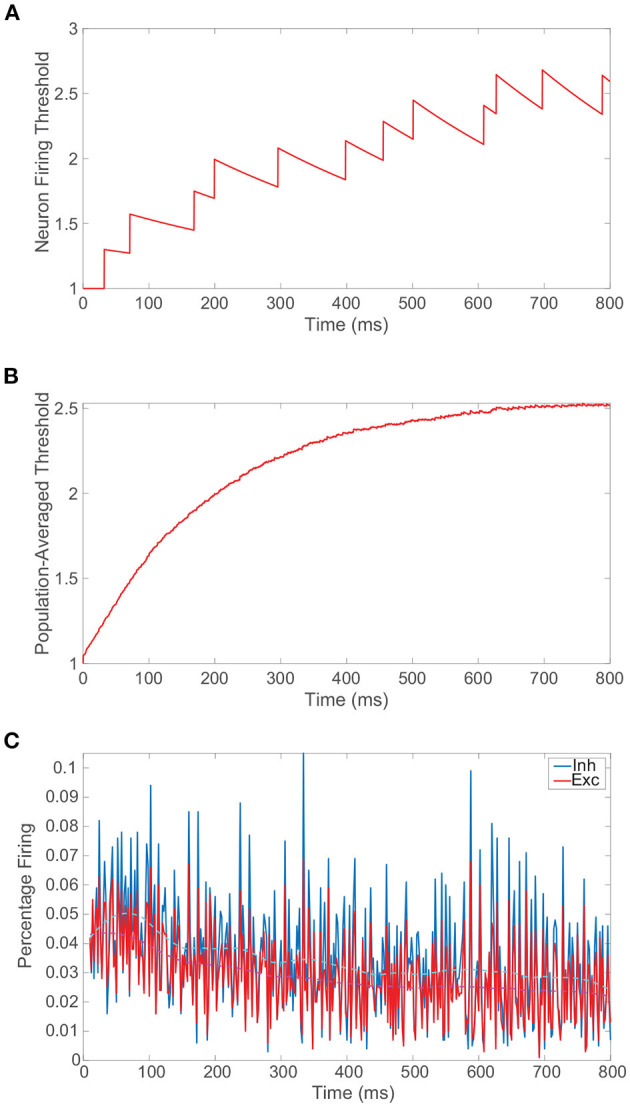
Balanced network dynamics with spike-frequency adaptation. **(A)** Firing threshold of a sample excitatory neuron over time in a balanced network incorporating spike-frequency adaptation. **(B)** Population-averaged firing threshold across the corresponding excitatory population over time. **(C)** The percentage of excitatory neurons firing (red) and inhibitory neurons firing (blue) as a function of time. A locally weighted moving average is plotted for the excitatory population in dashed magenta and for the inhibitory population in cyan. Adaptation parameters are λ = 0.05 and ϕ = 0.3, and the remaining parameters for the isolated balanced network considered are provided in Figure 1.

We conclude the preliminary discussion of our model network by examining the impact of the spike-frequency adaptation prescribed by Equation (1b). In Figure 2A, we plot the dynamics of the firing threshold for a sample neuron subject to moderately strong adaptation, demonstrating relatively large upward jumps in threshold when that neuron fires and a slow decay in the dynamic threshold between its spikes. The neuronal firing threshold shows a more rapid overall increase initially and then the threshold ultimately saturates at an elevated level once the neuron tends to spike more slowly. On a more global level, in Figure 2B we depict the population-averaged threshold dynamics with time for the excitatory neurons, which, albeit more smoothly, also exhibit a relatively rapid initial ascent and saturation in the long-time limit. Consequently, as seen in Figure 2C, the percentage of neurons firing decreases initially in time and then remains nearly constant with minor fluctuations about a now depressed mean. As demonstrated in prior work (Barranca et al., 2019), so long as the adaptation is not too strong, the network still demonstrates balanced dynamics with lower overall activity following an initial transient period. When spike-frequency adaptation is included only in the excitatory neurons, the parameter regime in which balanced dynamics are theoretically expected in fact broadens.

### 2.2. Binocular rivalry dynamics and model percept reconstructions

In order to reconstruct the dynamic percept during binocular rivalry, we derive a generalized input-output map capable of incorporating multiple stimuli in Section 4.1 and then construct a mechanism for selecting the appropriate percept at any given point in time. While previous work has developed such mappings in balanced networks using coarse-graining techniques rooted in statistical mechanics for I&F neurons without adaptation (Barranca, 2022) or binary neurons with adaptation (Barranca et al., 2019), the model in this work is more detailed and requires an extension of these methodologies. We note, however, that even if the network parameters are not strictly in the theoretical balanced operating regime, as long as the evoked dynamics are sufficiently irregular, our derivation of the input-output mapping still holds, making our framework robust to alternative model settings.

A typical requirement for balanced dynamics is that activity in both the excitatory and inhibitory populations remains non-zero and asynchronous in the large-network limit. Thus, we require the population-averaged firing rates in a given pool, _*m*_*z*_*k*_ for *k* = *E, I* and *z* = 1, 2, to obey 0 < _*m*_*z*_*k*_ < ∞ as *N* → ∞ and as *K* → ∞ given a fixed ratio *N*_*E*_/*N*_*I*_. In order to hold well for a finite-network realization, the linear input-output mapping and associated theoretical bounds on the model parameters require that the network is sufficiently large and composed of enough excitatory and inhibitory connections to generate sustained irregular activity.

**Figure 3:**
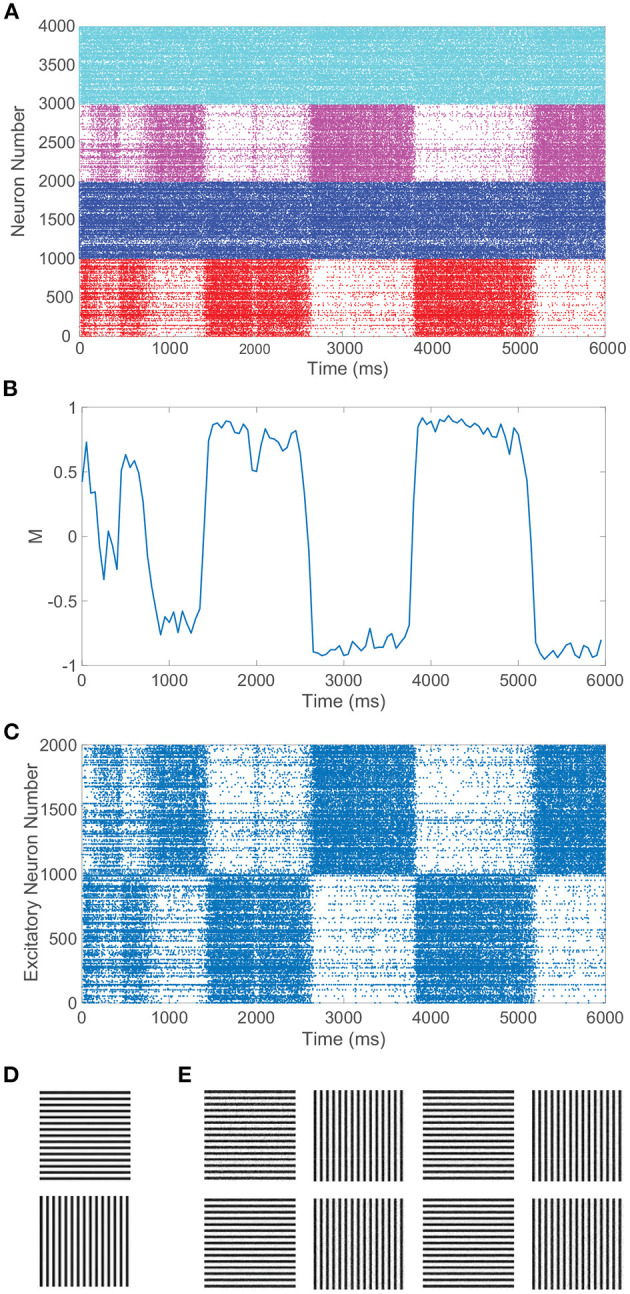
Rivalrous dynamics for network model with competing downstream pools and simple monocular image stimuli. **(A)** Raster plot exhibiting the firing times of all downstream neurons in the full model. Neurons in pool 1 are indexed 1 − 2000 (excitatory in red, inhibitory in blue) and neurons in pool 2 are indexed 2001 − 4000 (excitatory in magenta, inhibitory in cyan). The first 1, 000 neurons in a pool are excitatory. **(B)** Dominance metric, *M*, as a function of time given the model dynamics evoked by simple monocular image inputs. A value of 1 indicates that pool 1 is fully dominant and −1 indicates that pool 2 is fully dominant. **(C)** Raster plot for only the downstream *excitatory* neurons. Excitatory neurons in pool 1 are indexed 1 − 1000 and in pool 2 indexed 1001 − 2000. **(D)** 100 × 100 pixel grayscale stripes images driving the downstream neurons in pool 1 (top) and pool 2 (bottom). **(E)** Percept reconstructions from evoked network dynamics during each of the first eight dominance durations following the initial transient period (left to right). Parameters within each pool are identical to those in Figure 1 with λ = 0.00625, ϕ = 0.005, and long-range connection strength parameter *C*_*IE*_ = *R*_*IE*_ = 1.

In particular, we require balanced dynamics for neurons in the currently dominant pool, as excitatory neurons in the suppressed pool are expected to be significantly less active and more inhibited than assumed in a balanced network. Since the inter-pool connections are from excitatory to inhibitory neurons, the inhibitory neurons in the suppressed pool will typically continue firing from this additional cross-pool excitation while the excitatory neurons in the suppressed pool will primarily be quiescent. This is demonstrated in the raster plot in Figure 3A for a model network simulation of 6, 000 ms, showing persistent firing across both inhibitory populations for all time and significantly diminished firing activity across the excitatory neurons in the suppressed pool for each period of dominance. A relatively low level of firing in the non-dominant pool is observed in some experiments, consistent with the model dynamics (Blake and Logothetis, 2002), though whether the neurons firing in the suppressed pool are largely inhibitory in experiments is not yet fully determined.

The key input-output mapping, relating the monocular stimulus driving a downstream neuron in the first pool, **p_1_**, to the vectors of evoked neuronal firing rates and expected voltages across the downstream neurons in the first pool, **m_1_** and ${\bar{v}}_{1}$, respectively, may be expressed compactly across the network in matrix form as


(2) $${\bar{v}}_{1}=V^{Re}+\frac{1}{g_{L}}\left(F_{1}p_{1}+R_{1}m_{1}+C_{2}m_{2}-m_{1}\left({\bar{\theta }}_{1}-V^{Re}\right)\right),$$

where **R_1_** is ordered such that the first *N*_*E*_ rows (columns) correspond to the *N*_*E*_ excitatory neurons and the next *N*_*I*_ rows (columns) correspond to the *N*_*I*_ inhibitory neurons in pool 1; we assume the same ordering for the remaining terms in Equation (2). The time-averaged threshold of each neuron in the first downstream pool, ${\bar{\theta }}_{1}$, is included in the input-output mapping to account for the impact of spike-frequency adaptation, and the influence of inter-pool connections is accounted for by **C_2_m_2_**. Note that this mapping is valid when the first pool is dominant. The input-output mapping for downstream neurons in the second pool is similarly given by interchanging the numbered subscripts in Equation (2) and is analogously derived. This mapping assumes the periods of dominance are sufficiently long such that the dynamical statistics, **m_1_**, ${\bar{v}}_{1}$, and ${\bar{\theta }}_{1}$, are robust and well approximate long-time averages, which we have verified to be empirically true. In practice, statistics regarding the network dynamics are computed separately for each period of dominance and then separately used to reconstruct each corresponding dominant monocular stimulus.

Since the firing activity of the excitatory neurons is most significantly modulated across periods of dominance, the dominant stimulus at time *t* will be determined by the population-averaged firing rates of the excitatory neurons in first and second pools, _*m*_1_*E*_(*t*) and _*m*_2_*E*_(*t*), respectively, computed in a small time bin centered around time *t*. Based on these firing rates, we compute a time-varying dominance metric that prescribes both the dominant pool and the degree to which it currently dominates,


(3) $$\begin{array}{l} M\left(t\right)=\frac{{m_{1}}_{E}\left(t\right)-{m_{2}}_{E}\left(t\right)}{{m_{1}}_{E}\left(t\right)+{m_{2}}_{E}\left(t\right)}. \end{array}$$

The dominance metric varies between −1 and 1, where a value closer to 1 indicates stronger dominance of the first pool and a value closer to −1 indicates stronger dominance of the second pool. We assume a dominance period begins when *M*(*t*) switches sign and then remains above 0.4 in magnitude for at least 100ms.

Figure 3B depicts the dominance metric as a function of time for the same network simulation previously considered in Figure 3A. Here, we observe an initial transient period in which neither stimulus is fully dominant. Then we subsequently see clear alternating periods of dominance for each pool, with metric values hovering near −1 or 1, along with fast transitions in the dominance metric value at the end of each period of dominance. As will be described in more detail in the next section, these dominance durations agree well with experimental observations and demonstrate irregularity in length. Note that the dynamics of the dominance metric specifically correspond to the rivalrous alternations in excitatory neuron firing activity shown for this simulation in Figure 3C, determining the duration of each dominance period, the rapid transitions in dominance, and the degree to which a given pool is dominant. In this case, the respective monocular stimuli driving the neurons in the two downstream pools are simple grayscale horizontal and vertical gratings, depicted in Figure 3D.

With a means of identifying the dominant stimulus as well as an input-output mapping, what remains to be determined is a method for reconstructing the appropriate percept given measurements of network dynamics during a period of dominance. In order to apply Equation (2) and recover the percept, only the monocular stimuli are assumed unknown and all remaining terms must either be known or estimated. The connectivity matrices are known exactly following a particular network realization and fixed model parameters, *g*_*L*_ and *V*^*Re*^, are known as well.

Due to the assumed compressive nature of the feedforward pathway, i.e., since there are many more monocular stimulus components (pixels) than downstream neurons, reconstructing the dominant stimulus during each dominance duration from our input-output mapping requires solving a highly underdetermined linear system with infinitely many solutions. Addressing this issue, to select the percept most closely associated with the true dominant monocular stimulus, we apply compressive sensing (CS) theory to linear system (2). CS theory is a modern mathematical breakthrough that provides a means of efficiently sampling and reconstructing signals which are sparse in an appropriately-chosen domain (Candes et al., 2006; Donoho, 2006; Candes and Wakin, 2008), and it has promoted discoveries in diverse applications across physics, biology, and image processing (Lustig et al., 2007; Bobin et al., 2008; Dai et al., 2009; Herman and Strohmer, 2009; Berger et al., 2010; Gross et al., 2010; Wang et al., 2011; Noor et al., 2013; Emad and Milenkovic, 2014). Natural scenes and gratings, common as monocular inputs in binocular rivalry, are known to have sparse representations in frequency-based spaces (Field, 1994) and thus CS techniques are amenable to their reconstructions. As discussed in detail in Section 4.2, compressive sensing reconstructions generally require *linear* measurements of *static* sampled data (Candes et al., 2006; Donoho, 2006), but our model demonstrates non-linear dynamics in time. The derived input-output mapping therefore allows us to successfully overcome this conceptual obstacle by approximating a linear relationship between the monocular stimuli and limited observations of the evoked network dynamics, facilitating efficient reconstructions of the dominant stimuli.

We use the network model output over a given dominance duration to estimate the terms reflecting dynamical statistics in Equation (2) and then apply a CS recovery algorithm known as the orthogonal matching pursuit (Tropp and Gilbert, 2007) to reconstruct the dominant stimulus. For a given period of dominance, we record the firing rates, time-averaged voltages, and time-averaged thresholds across all downstream neurons to obtain estimates for **m_*z*_**, ${\bar{v}}_{z}$, and ${\bar{\theta }}_{z}$, respectively. When the metric abruptly switches sign and signals an alternation, we stop collecting output data and perform a reconstruction. In the recovery process when the first pool is dominant, the stimulus **p_1_** is considered the unknown input in the input-output mapping given by Equation (2), and the remaining terms are either known model parameters or measured from model simulation. In this case, the feedforward connectivity matrix **F_1_** plays the role of the CS measurement matrix, and the solution obtained *via* the orthogonal matching pursuit, **p^recon^**, corresponds to the percept and is considered optimal when it is close to the true dominant stimulus **p_1_**. After this reconstruction, we begin to recollect new output data throughout the next dominance duration for the subsequent reconstruction; this process of reconstructing and recollecting data continues throughout the simulation, giving a sequence of estimates for the percepts during the rivalrous alternations.

CS reconstructions of the switching percept using the network dynamics during each of the first eight dominance durations following the initial transient period are given in Figure 3E. In each case, the reconstruction is nearly perfect, showing successful encoding of the dominant percept in the downstream dynamics. We emphasize that there is minimal information loss from the non-linear dynamics and downstream compression for these relatively simple images. Note that the pixel values are normalized so that each monocular stimulus has the same average pixel value and the feedforward drive from the two input images is comparable. As a result, there is little systematic difference in both the dominance durations and the reconstruction quality corresponding to the two monocular stimuli.

It is important to emphasize that while it may be possible for the brain to implement CS recovery in general (Rozell et al., 2008), we make no claim that our precise method of reconstructing the percept is performed by the visual system itself and hypothesize that summation implemented by a downstream binocular neuronal network or dichoptic differencing of monocular input *via* opponent neurons (Lansing, 1964; Bock et al., 2019) may be able to compute a dominance metric in the determination of the dynamic percept. Nonetheless, we see that rivalrous behavior can manifest even in the presence of potential information loss through a downstream sensory bottleneck and that monocular image information is well-encoded in the dynamics of the downstream neurons in the dominant pool. Even if network dynamics data is instead only collected during the first 200 ms of a given dominance period rather than throughout the entire dominance duration, we have verified that there is little change in the percept reconstruction quality and thus monocular stimulus information is indeed quite rapidly encoded.

**Figure 4:**
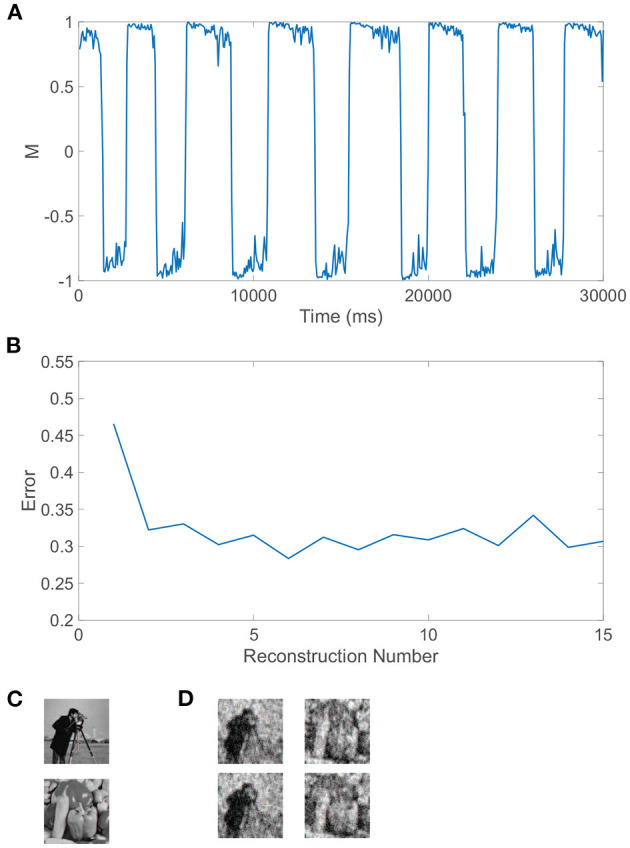
Rivalrous dynamics for network model with competing downstream pools and natural scene monocular stimuli. **(A)** Dominance metric, *M*, as a function of time given the model dynamics evoked by natural scene monocular image inputs. **(B)** Relative reconstruction error for percept reconstructions using downstream network dynamics during each dominance duration in **(A)** as time increases. **(C)** 100 × 100 pixel grayscale natural scene images driving downstream neurons in pool 1 (top) and pool 2 (bottom). **(D)** Percept reconstructions from the evoked network dynamics during each of the first four dominance durations following the initial transient period (left to right). Parameters are identical to those in Figure 3.

We can similarly consider the model dynamics evoked when the two monocular stimuli are more complicated natural scenes, particularly those depicted in Figure 4C. In this case, we again observe clear alternations in the dominance metric plotted in Figure 4A, hovering near a value of 1 and then rapidly switching to gravitate near −1, with such alternations continuing to occur irregularly throughout time. Each dominant monocular stimulus is analogously reconstructed *via* Equation (2) using data collected during each dominance duration. To measure the accuracy of a given stimulus reconstruction, **p^recon^**, we compute the relative reconstruction error, ∥**p**−**p^recon^**∥/∥**p**∥, using the Euclidean norm, $\|p\|=\sqrt{\sum_{i}p_{i}^{2}}$ and true dominant stimulus **p**.

The relative error for each monocular natural scene reconstruction is plotted in Figure 4B over all dominance periods in the model simulation. Here, we include as the first data point the relative reconstruction error using network dynamics collected over the initial transient period, where the metric initially hovers slightly below 1 for a time shorter than a typical dominance duration. As expected from experimental results (Blake et al., 1991; Pearson and Brascamp, 2008), the reconstruction error in the transient period is relatively high and likely corresponds to the mixed percept often reported initially in binocular rivalry experiments. The relative reconstruction errors corresponding to each of the subsequent dominance periods are lower and display only minor fluctuations in value. We provide several sample percept reconstructions using data collected during each of the dominance durations following the initial transient period in Figure 4D. The reconstructions are each recognizable though less accurate than those obtained for the grating images considered previously. This is to be expected since these natural scenes display more complex structure in the sparse frequency space and yet only contain 10, 000 pixels. For higher resolution images with more pixels and consequently more sparsity in the frequency domain, more accurate CS reconstructions are generally achievable given the same ratio of input image pixels to downstream neurons (Barranca et al., 2016). While high-frequency components are added when the resolution of an image is increased, the amplitude distribution of the dominant low-frequency components is often nearly identical to that of a lower resolution version of the image, and the additional downstream neurons introduced to maintain the same factor of compression will generally increase the accuracy of stimulus recovery since the nearly fixed dominant frequency-components become better resolved when more neurons are available to sample them.

### 2.3. Dominance duration distribution and robustness to alternative architectures

Widespread experimental studies in both humans and non-human mammals indicate that dominance duration distributions in binocular rivalry are largely peaked, right-skewed, a few seconds in length, and conform well to a gamma distribution (Kovacs et al., 1996; Leopold and Logothetis, 1996). Due to their ubiquity and ease of application, such observations often serve as natural benchmarks for physiologically reasonable models of binocular rivalry. From the dynamics of the dominance metric displayed in Figures 3, 4, we see that model dominance durations are indeed often between 1 and 2 s with irregularity in their specific lengths.

**Figure 5:**
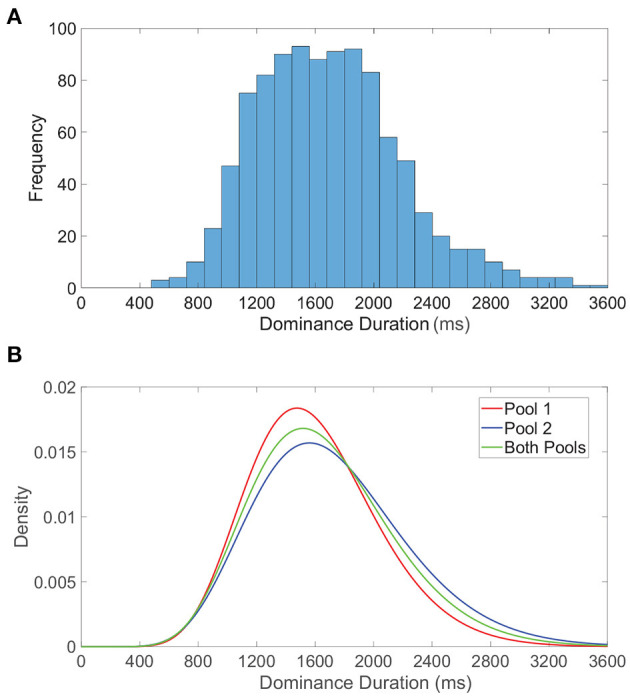
Dominance duration distributions. **(A)** Histogram of all dominance durations across 50 network realizations each with duration 40, 000 ms. **(B)** Gamma distributions fitted to the dominance duration histograms for pool 1 (red), pool 2 (blue), and both pools (green). For the fitted gamma distributions, the pool 1 shape parameter is 12.7176 and scale parameter is 125.942, the pool 2 shape parameter is 10.5895 and scale parameter is 162.762, and for both pools the shape parameter is 11.3812 and scale parameter is 146.096. Network model parameters and monocular image inputs are identical to those in Figure 3.

We examine the distribution of dominance durations in more detail in Figure 5A, where we plot a histogram of all dominance durations for 50 model network realizations each with a total runtime of 40 s. This is meant to reflect the typical experimental setting in which rivalry dynamics are analyzed for multiple individuals (network realizations) for a relatively long time course. The resultant histogram is right-skewed with mean near 2 s, conforming to classical dominance duration statistics. In Figure 5B, we similarly examine the dominance duration distributions for the first pool, second pool, and both pools, fitting each to a gamma distribution. Separately, the dominance durations in each pool are also well-approximated by a gamma distribution, with slight shifting in mean likely as a consequence of differences in feedforward connectivity and monocular input stimuli.

We note these peaked gamma distributions indicate that the probability of a subsequent switch immediately following an alternation is low, giving further credence to the notion that sufficient adaptation must accumulate, or alternatively wear off, before another percept switch can occur. It is important to emphasize that while dominance durations for a specific network realization are quite irregular, there is still more correlation in dominance durations for a particular network than across distinct network realizations. The inclusion of data accumulated over multiple network realizations facilitates the especially long right tail in the dominance duration distributions, corresponding primarily to those network realizations that tend to produce especially long periods of dominance. This is consistent with the marked variations in average dominance duration across different individuals in experiments (Gallagher and Arnold, 2014; Bosten et al., 2015; Bock et al., 2019), and can potentially be attributed to underlying genetic or chemical differences (Klink et al., 2010; Miller et al., 2010; Shannon et al., 2011). It is worth noting that some studies report a correlation between sequential dominance durations in certain experimental settings (van Ee, 2009), especially demonstrating history dependence after prolonged exposure to a non-ambiguous display and highlighting the possible role of adaptation (Nawrot and Blake, 1989). While we do not explicitly consider how such correlations may be modulated or manifest, one approach to examining history dependence in the context of our model would be to include white noise in the dynamic firing threshold activity and examine its potential affect on serial correlations.

**Figure 6:**
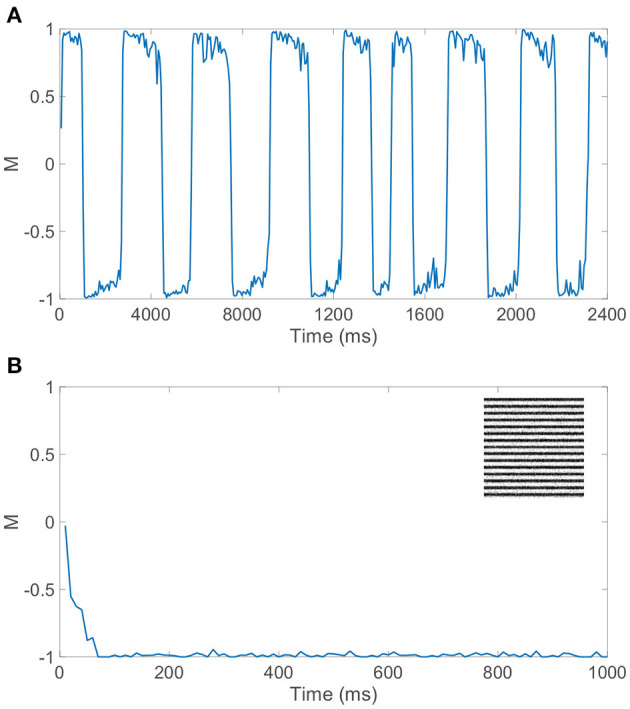
Dynamics for network model with competing downstream pools given alternative choices of spike-frequency adaptation. **(A)** Dominance metric, *M*, as a function of time when only the excitatory neurons in each pool undergo spike-frequency adaptation. **(B)** Dominance metric, *M*, as a function of time when spike-frequency adaptation is removed entirely from the model, indicating the rapid and sustained dominance of pool 2 for this network realization. Inset: Monocular stimulus reconstruction from the corresponding dynamics of pool 2. Network model parameters and monocular image inputs are otherwise identical to those in Figure 3.

Gamma-distributed dominance durations and rivalrous switching are present for a relatively broad set of model parameters and ratios of excitatory to inhibitory neurons as long as the dynamics within the dominant pool are largely balanced. While the majority of our model simulations utilize a 1:1 ratio of excitatory to inhibitory neurons to ensure sufficiently well-balanced dynamics for a given finite network realization, we note that similar switching dynamics are observed when a 4:1 ratio of excitatory to inhibitory neurons is utilized consistent with estimates in V1 (Gilbert, 1992; Liu, 2004). Moreover, if spike-frequency adaptation is only incorporated in excitatory neurons, holding the firing thresholds of all inhibitory neurons at θ_*I*_, realistic rivalrous activity is also produced as depicted by the dominance metric dynamics in Figure 6A. Experimental evidence indicates that excitatory neurons are generally more likely to undergo adaptation than inhibitory neurons (La Camera et al., 2006; Augustin et al., 2013), and we see that binocular rivalry is consistent with this network setting as well. In fact, prior theoretical analysis demonstrates that the presence of spike-frequency adaptation only in the excitatory population can broaden the space of model parameters over which balanced dynamics manifest (Barranca et al., 2019).

If instead spike-frequency adaptation is removed from our model entirely, by fixing all excitatory neuron firing thresholds at θ_*E*_ and all inhibitory neuron firing thresholds at θ_*I*_, rivalrous dynamics are not produced and there is a single dominant pool throughout time. As depicted in Figure 6B, the dominance metric rapidly gravitates toward 1 or −1, remaining near that value perpetually and failing to produce any switching even over a long time horizon. The identity of the dominant pool is random and dependent on the specific network realization, with balanced dynamics in the dominant pool as seen during a specific dominance period in the full model. This underlines the key role of a fatigue mechanism in binocular rivalry, and without irregularity in the model dynamics, which is produced under the balanced network paradigm in this case, the ubiquitous skewed dominance duration distribution is eliminated. Thus, we provide evidence for the necessity of both irregular network dynamics and slow adaptation, beyond the competition fostered by long-range connections between the downstream pools, to fully produce the stochastically switching percept that is the cornerstone of binocular rivalry.

### 2.4. Levelt's laws and the impact of stimulus strength on alternations

Levelt's four laws often provide a gold standard on which models of binocular rivalry can be tested for biological realism and applied to discover general principles for visual perception, summarizing the core relationships between variations in stimulus characteristics and resultant rivalrous dynamics (Levelt, 1965). These propositions have evolved into a more modern form over the last half century, accumulating new insights over a wide array of modern experiments that are summarized in (Brascamp et al., 2015), and we address each in turn using our model network dynamics. While experimental studies involving Levelt's laws typically consider the impact of stimulus strength based on the relative contrast, density, or blur of contours in each monocular stimulus for relatively simple images, in our model we consider the stimulus strength to be directly determined by the mean feedforward input for a downstream neuron. While there is not a single notion of stimulus strength across all experiments, meaning alternative perturbations in the stimuli can predictably effect rivalrous dynamics, our choice allows us to precisely probe the strength of any stimulus in a unified manner, including complex natural scenes as well as simple gratings.

**Figure 7:**
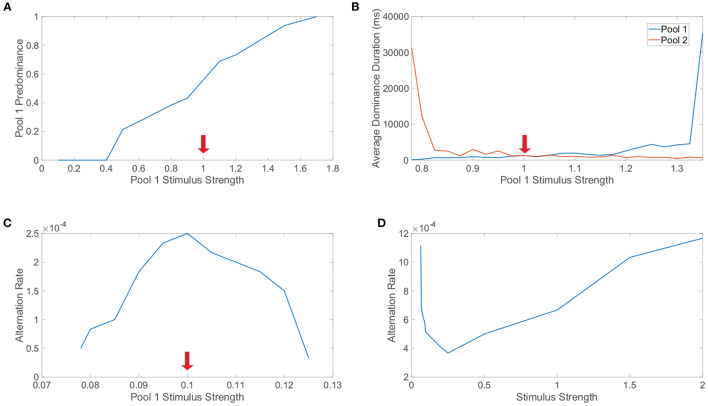
Levelt's laws and network model dynamics. **(A)** Investigation of Levelt's first law. The stimulus strength of pool 1 is varied while the stimulus strength of pool 2 is fixed at 1 (indicated by red arrow). For each choice of stimulus strength, the predominance of pool 1 is plotted, indicating the proportion of time in which pool 1 is dominant. **(B)** Investigation of Levelt's second law. Stimulus strengths are as in **(A)** and now the average dominance durations for pool 1 (blue) and pool 2 (red) are plotted. **(C)** Investigation of Levelt's third law. The stimulus strength of pool 1 is varied while the stimulus strength of pool 2 is fixed at 0.1. The alternation rate for pool 1 is plotted for each choice of stimulus strength, indicating the number of dominance durations for pool 1 per unit time (ms). **(D)** Investigation of Levelt's fourth law. The stimulus strengths of both pool 1 and pool 2 are varied together. The alternation rate summed across both pools is plotted for each choice of stimulus strength, including switches from either monocular stimulus. Network model parameters and monocular image inputs are otherwise identical to those in Figure 3 for a simulation time of 40, 000 ms.

Levelt's first law posits that *increasing the strength of one monocular stimulus only will increase the predominance of that particular monocular stimulus*. Note that the predominance of a specific monocular stimulus is the proportion of viewing (simulation) time in which that monocular stimulus is dominant. In Figure 7A, we explore Levelt's first law by varying the monocular stimulus strength, *m*_0_, for the first pool only and fixing the monocular stimulus strength for the second pool. Across a sequence of such simulations, we plot the predominance of the first pool, which indeed increases with its monocular stimulus strength for sufficiently large feedforward drive; this trend holds over a range of *m*_0_ values that broadly surrounds the fixed monocular stimulus strength for the second pool. Intuitively, Levelt's first law agrees with the idea that the pool with the stronger monocular stimulus should be dominant more often since it is predisposed to suppressing the competing pool.

Proceeding in turn, Levelt's second law postulates that *increasing the difference between the two monocular stimulus strengths will primarily increase the average dominance duration for the percept corresponding to the stronger monocular stimulus strength*. We investigate Levelt's second law in Figure 7B by again varying the monocular stimulus strength for only the first pool and now plotting the resultant average dominance durations for each pool across the corresponding simulations. We observe that when the second pool has a stronger monocular stimulus and the monocular stimulus strength for the first pool is decreased, the average dominance duration for the second pool markedly increases with minimal change in the average dominance duration for the first pool. Likewise, when the stimulus strength for the second pool is less than the stimulus strength for the first pool, as the stimulus strength for the first pool is increased, the average dominance duration for the first pool increases with only a minor change in the average dominance duration for the second pool. Together, these observations conform precisely to the prescription provided by Levelt's second law.

Levelt's third law proposes that *increasing the difference between the two monocular stimulus strengths will reduce the alternation rate*. Note that here the alternation rate refers to the total number of perceptual switches that occur per unit time. We explore Levelt's third law in Figure 7C by once again varying the monocular stimulus strength for the first pool while fixing that of the second pool, plotting the alternation rate in each case. As expected from Levelt's third proposition, the highest alternation rate occurs when the two monocular stimuli are of equal strength, with consistently decreasing alternation rate as the first pool stimulus strength is either further increased or decreased from the fixed second pool stimulus strength. One can argue that Levelt's third law is in fact equivalent to Levelt's second law, since when the average dominance durations for the two pools in sum are lowest, there will generally be the most alternations. However, the two laws are still often separated because they observe this shift in dynamics with a slightly different perspective and were more distinct in the context of the original non-updated version of Levelt's laws.

Finally, Levelt's fourth law asserts that *increasing the monocular stimulus strengths of each eye together, such that the two monocular stimulus strengths are equal in each case, will generally increase the alternation rate, except at particularly low monocular stimulus strengths for which this trend is reversed*. To probe this final law, we vary together the monocular stimulus strengths for each pool, so the two downstream pools have the same average feedforward drive in a given trial, and plot the alternation rate for each choice of monocular stimulus strength in Figure 7D. We see that as the stimulus strength is increased beyond *m*_0_ = 0.25, the alternation rate monotonically increases. On the other hand, for low stimulus strengths, we note that as the stimulus strength is instead decreased below 0.25, the alteration rate increases. These results fully agree with this modern form of Levelt's fourth law, including the more subtle shift in trend that occurs for low stimulus strengths. While the majority of studies demonstrate an increase in alternation rate with increasing stimulus strength, several model-driven explorations (Shpiro et al., 2009; Seely and Chow, 2011) as well as select experiments (Platonov and Goossens, 2013) alternatively exhibit an increase in alternation rate as stimulus strength is decreased over a range of weak strengths. It is likely that the regime of small stimulus strengths for which this reversal occurs is difficult to produce in experiments for specific notions of stimulus strength, causing most experimental settings to more naturally indicate that alternation rate increases with stimulus strength.

**Figure 8:**
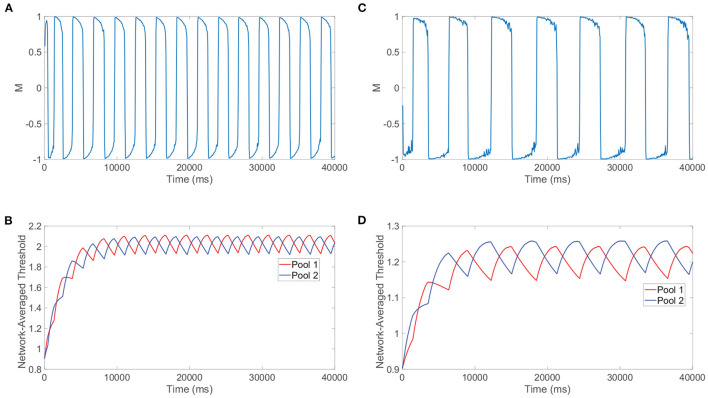
Mechanisms for switching phenomenon. **(A)** Dominance metric, *M*, as a function of time when *m*_0_ = 1 and the alternation rate is high. **(B)** Network-averaged firing threshold across all downstream neurons in pool 1 (red) and pool 2 (blue) over time for *m*_0_ = 1. **(C)** Dominance metric, *M*, as a function of time when *m*_0_ = 0.25 and the alternation rate is low. **(D)** Network-averaged firing threshold across all downstream neurons in pool 1 (red) and pool 2 (blue) over time for *m*_0_ = 0.25. Network model parameters and monocular image inputs are otherwise identical to those in Figure 3.

Beyond reproducing experimental observations, Levelt's fourth law and our related model analysis together support concrete mechanisms for the percept switching that depend on the strength of the stimuli. To demonstrate this, we compare the model network dynamics for two different stimulus strengths, with one in the classical Levelt's fourth law regime and the other in the reversal regime. We plot in Figure 8A the dominance metric dynamics for a relatively large stimulus strength of *m*_0_ = 1 and depict the corresponding dynamics of the network-averaged thresholds for the two pools in Figure 8B. We do the same in Figures 8C, D, respectively, when the two downstream pools are each driven using a particularly small stimulus strength of *m*_0_ = 0.25. As expected from Levelt's fourth law and Figure 7D, the dominance durations are generally longer when the stimulus strength is relatively small.

More informative to explaining the reversal at small stimulus strengths, however, is the difference in the saturation of the network-averaged thresholds in the two cases. For relatively high *m*_0_, it can be argued that the suppressed pool is responsible for the percept switch in what is often called the escape mechanism (Brascamp et al., 2015). In this case, decreasing the external drive strength results in longer periods of dominance (lower alternation rate) because generally the firing thresholds for neurons in the suppressed pool will need to drop yet lower to produce spikes and actively escape domination. As *m*_0_ is decreased, the suppressed pool has less total external drive, so the maximal voltage that a constituent neuron can achieve through fluctuations in its input current will be lower and hence the dynamic threshold will need to decrease further over the course of more time for a spike to be viable. In the regime of high *m*_0_, as exemplified by the steeply decreasing thresholds in Figure 8B, a necessary fixed decrease in firing threshold is achieved quite rapidly since the firing thresholds of the suppressed pool are relatively high initially and thus the first derivatives in threshold are generally large in magnitude in accordance with Equation (1b).

On the other hand, for small *m*_0_ below 0.25, it can be argued that the dominant pool is instead responsible for the percept switch in what is often called the release mechanism. Here, decreasing the external drive strength results in shorter periods of dominance (higher alternation rate) because generally the firing thresholds for neurons in the dominant pool will need to rise less for the spiking of the dominant neurons to be extinguished and thereby release the suppressed pool from domination. Decreasing the external drive results in shorter dominance durations because neurons in the dominant pool become unable to spike for a lower firing threshold that will take less time to reach *via* spike-frequency adaptation. This alternative mechanism corresponds to the relatively shallow declines in firing threshold exhibited in Figure 8D, since for sufficiently small *m*_0_ the amount of time necessary for the suppressed pool thresholds to recover (escape) becomes excessively long and what instead begins to happen first is the thresholds of neurons in the dominant pool become so high that spiking is no longer feasible. From the perspective of Equation (1b), a necessary fixed decrease in threshold for neurons in the suppressed pool is achieved very slowly since the firing thresholds of constituent neurons are now relatively low and thus the first derivatives in threshold are typically small in magnitude; therefore, the mechanism changes and the dominant neurons' inability to fire largely drives the percept switching for sufficiently small *m*_0_. We note that the exact time of the switch for either mechanism is random due to the fluctuations in neuronal inputs and irregular dynamics in the balanced regime, and when the approach of the firing threshold toward a critical value necessary for switching is especially slow in the case of the long dominance durations for *m*_0_ = 0.25, there is a larger degree of variability in the dominance durations. Such a shift from the escape to release mechanism observed empirically in our detailed neuronal model is indeed carefully documented and substantiated by prior theoretical studies (Curtu et al., 2008), where rigorous analytical techniques and dynamical systems theory were utilized in the context of a two-population firing rate model to characterize the change in mechanism for low stimulation strengths.

### 2.5. Rivalry dynamics and receptive field structure

While up to this point we have demonstrated the successful dynamic encoding of monocular stimulus percepts during binocular rivalry and the adherence of our model activity to canonical experimental constraints, we now turn to show that these results are robust to more biologically faithful choices of feedforward connectivity beyond the uniformly random structure assumed in the prior sections. In particular, receptive field structure in feedforward connectivity is ubiquitous throughout much of the visual system in the sense that downstream neurons are most stimulated by a range of stimuli with analogous features (Welker, 1976; Graziano and Gross, 1993; Wilson, 2001). This includes neurons in the retina, LGN, and V1, though the precise prototypical features of the receptive fields vary by brain area. In the LGN, for instance, neurons generally possess center-surround receptive fields, aggregating the output of upstream ganglion cells that together sample a spatially localized region of visual space, with inputs from the central circle and surrounding annulus of the receptive field having opposite (excitatory or inhibitory) effect (Hubel and Wiesel, 1960; Wiesel, 1960). In this case, the overall size of the spatial receptive field determines the spatial frequencies in visual space that are encoded and the center-surround structure facilitates the encoding of image edge information.

Prior work in the context of a single stimulus driving both eyes has showed that, compared to the uniformly random feedforward connectivity initially assumed in our two-layer model, spatially localized feedforward connectivity inspired by receptive fields can more accurately encode the dominant low and moderate frequency components composing most natural stimuli (Barranca et al., 2016). We focus next on the spatially localized nature of these receptive fields for simplicity and generality, noting that the inclusion of center-surround antagonism does not significantly alter the compressed encoding of images in the downstream network dynamics and still adheres to the requirements of compressive sensing theory if sufficient randomness is incorporated (Barranca and Zhu, 2018). To include spatial structure, we assume that a pixel in *n*-vector **p_z_** is mapped to a distinct (*x, y*) location with integer coordinates on a $\left[1,\;\sqrt{n}\right]\times \left[1,\;\sqrt{n}\right]$ Cartesian grid corresponding to its row and column location in the pixel matrix that produced vectorization **p_z_**. Each row of the feedforward connectivity matrices is then mapped to a distinct random location (x_i_, y_i_) on this grid, around which the receptive field of the *i*^th^ downstream neuron is centered. Incorporating both spatial localization and randomness in the spatial receptive fields, we assume the probability, *P*, that the *i*^th^ downstream neuron samples a pixel with spatial coordinates (*x*_*j*_, *y*_*j*_) is given by


(4) $$\begin{array}{l} P=\rho e^{-\left[{\left(x_{i}-x_{j}\right)}^{2}+{\left(y_{i}-y_{j}\right)}^{2}\right]/2\sigma^{2}}, \end{array}$$

where ρ is the sampling probability if (*x*_*i*_, *y*_*i*_) = (*x*_*j*_, *y*_*j*_), when the receptive field is centered at the location of a given pixel, and σ scales the spatial size of the receptive field, which is known to vary throughout the visual system (Hubel, 1995; Sceniak et al., 1999). Therefore, each feedforward connection is prescribed by a Bernoulli random variable, determined independently of all other connections, with success probability given by Equation (4).

**Figure 9:**
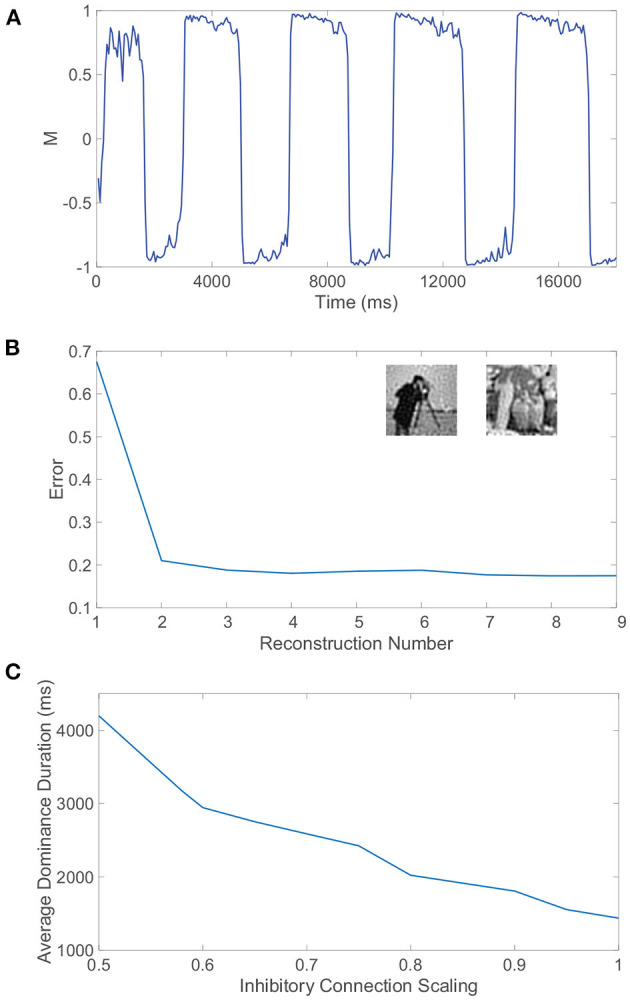
Receptive field structure, autism, and rivalrous dynamics. **(A)** Dominance metric, *M*, as a function of time when the feedforward connectivity matrices both have spatially localized receptive field structure prescribed by Equation (4) with parameters ρ = 0.92 and σ = 2.2. **(B)** Relative reconstruction error for percept reconstructions using evoked network dynamics during each dominance duration in **(A)** as time increases. Inset: Monocular image input reconstructions from the dynamics of pool 1 and pool 2, respectively, during two subsequent dominance durations. **(C)** Average dominance duration as a function of the inhibition scaling factor, multiplying all inhibitory connections in each pool, computed over a simulation time of 40, 000 ms for each scaling. Network model parameters and monocular image inputs are otherwise identical to those in Figure 4.

We utilize the (ρ, σ) parameter choice shown previously to optimally encode a single 100 × 100 pixel natural scene stimulus in a single-pool network model. The generated feedforward connectivity matrices are sparse with connection density near 0.001, where σ = 2.2 produces moderately-sized receptive fields and ρ = 0.92 allows each pixel to be sampled on average at least one time (Barranca et al., 2016). The resultant dominance metric dynamics are plotted in Figure 9A, which demonstrate clear rivalrous switching behavior and largely resemble the activity produced in the case of the uniformly random feedforward connectivity considered in the previous sections. What does change, however, is the quality of the monocular natural scene percept reconstructions during each period of dominance, as evidenced in Figure 9B. While there still exists an initial transient period in which neither stimulus is well-encoded, reconstructions using data from the subsequent dominance durations are significantly improved relative to those produced for the natural scenes considered in Figure 4B with uniformly random feedforward connectivity. The relative reconstruction errors are nearly halved, gravitating around a constant low value.

Sample reconstructions of the corresponding two monocular natural scene inputs are depicted in the inset of Figure 9B, visually demonstrating quite accurate encoding. Hence, the proposed mechanisms for binocular rivalry are robust to more realistic feedforward network architectures that are largely absent from prior model investigations. The continued presence of the initial transient period indicates that its entrenchment is beyond the feedforward connectivity structure, and the associated mixed percepts may in fact manifest as a result of initial information loss due to the sensory bottleneck or an intrinsic lag in processing *via* the non-linear network dynamics.

### 2.6. Relationship between autism and binocular rivalry

The excitation/inhibition imbalance hypothesis suggests that levels of excitation and inhibition are abnormal throughout many brain areas for individuals with autism (Rubenstein and Merzenich, 2003; Jamain et al., 2008), but clear markers for this imbalance in the autistic brain are still needed. At the same time, several experimental studies indicate that inhibition levels in the brain may be implicated in variations in both alternation rate and the prevalence of mixed percepts in binocular rivalry (Robertson et al., 2013; Dieter et al., 2017; Spiegel et al., 2019). Alternation rate may therefore serve as a non-invasive indicator of autism, and in light of evidence that the autistic brain exhibits reduced inhibition due to impaired GABA signaling (Robertson et al., 2016; Mentch et al., 2019), we explore the effect of reduced inhibition in our model on rivalrous dynamics.

To adjust the level of inhibition, we multiply all inhibitory connection strengths in our model, particularly parameters *R*_*EI*_ and *R*_*II*_, by a scaling factor *S*_*I*_. For each choice of inhibitory scaling factor, we record the dynamics of our model and compute the average dominance duration, plotting the result in Figure 9C. We observe that as the inhibition in the model decreases, the average dominance duration increases and the alternation rate correspondingly decreases. This monotonic trend strongly suggests that binocular rivalry is indeed weakened as a result of the reduced inhibition often associated with autism. In fact, if the inhibition strength is yet weaker than considered in Figure 9C, we begin to see trials in which either full winner-take-all dynamics manifest with no percept switching or interspersed long periods of time in which the dominance metric is near 0 indicative of a mixed percept, which is consistent with the longer periods of mixed percepts reported for autistic individuals in experiments (Robertson et al., 2013).

It should be noted that the conditions for balanced dynamics in an isolated (or highly dominant) pool given by Equation (9) are still met under this scaling of inhibition, which has no impact on the quotient $\frac{\left|R_{EI}\right|}{\left|R_{II}\right|}$ in the inequality, and thus the diminished rivalry is theoretically not a direct result of balanced dynamics breaking down. It is potentially the case that the reduced inhibition results in weaker competition between the downstream pools and consequently the slow adaptation dynamics must evolve over a longer time scale to compensate. One possible explanation according to Equation (8) is that the inhibitory neurons are expected to fire at a higher rate when their synaptic influence is weakened; so immediately following an alternation, the elevated firing thresholds of the inhibitory neurons in the newly suppressed pool will need to fall more to reach a fixed critical threshold for which a percept switch can occur and as a result more time will elapse until the next alternation.

## 3. Discussion

In the context of our newly formulated two-layer neuronal network model, we develop a framework for estimating the dynamic percepts in binocular rivalry based on explicit monocular stimulus reconstructions using measurements of network dynamics collected during each period of dominance. Our model suggests that to generate realistic rivalrous alternations it is sufficient to include irregular neuronal activity rooted in balanced network dynamics, competition between the downstream pools mediated by long-range connections from excitatory neurons in one pool to inhibitory neurons in the other, and slow fatigue *via* spike-frequency adaptation, showing that the removal of any one of these characteristics eliminates the dynamical features key to binocular rivalry. Moreover, beyond its novelty in the ability to directly incorporate natural scene monocular stimuli, our rivalry model is among the first to include complex network structure, such as feedforward connectivity and downstream compression, and uniquely produces distinct dynamics during the early transient period, which may correspond to the initial mixed percepts widely observed in experiments. We resolve the potential sensory bottleneck, where photoreceptor output is processed by the activity of smaller networks of neurons in downstream layers, by deriving an approximate linear input-output map embedded in the non-linear network dynamics and then utilizing compressive sensing theory to accurately recover a sequence of dominant monocular stimuli corresponding to the dynamic percepts. Incorporating additional facets of visual system structure observed empirically, such as spatial localization in receptive fields and adaptation present in excitatory neurons only, we underline the robustness of the proposed mechanisms for rivalrous dynamics and demonstrate strong agreement with canonical experimental findings that serve as litmus tests for rivalry models, such as Levelt's laws and gamma-distributed dominance durations.

While our study does address many features novel to theoretical investigations of binocular rivalry, experimental observations have unearthed additional factors that may play important roles in the phenomenon, such as top-down feedback from higher cortical areas and attention (Brown and Norcia, 1997; Zhang et al., 2011; Dieter et al., 2016; Li et al., 2017), and their dynamics may significantly modulate the lower-level processing primarily considered in this work. The primary visual cortex, as examined in our model, is the classical area implicated in generating perceptual bistability through what is termed eye-based rivalry, arising from competition between neurons driven by monocular inputs. While eye-based rivalry is well-supported experimentally (Xu et al., 2016), there is compelling evidence of stimulus-based rivalry, where the dynamics of binocular and feature-selective neurons in higher visual system areas are instead associated with perceptual changes in binocular rivalry (Leopold and Logothetis, 1996). While we largely remain agnostic on this issue, it may be the case that the two proposed types of rivalry together yield a more complete explanation of the true phenomenon (Lee and Blake, 1999; Tong, 2001).

Beyond the bistability examined in this work, alternative perceptual behaviors closely related to binocular rivalry are possible and may be studied using analogous techniques. Multistable rivalry between more than two percepts can manifest when parts of the two monocular stimuli may be divided and recombined into various coherent images, generating perceptual alternations between the original monocular stimuli as well as the meaningfully regrouped coherent images (Kovacs et al., 1996; Suzuki and Grabowecky, 2002; Sterzer et al., 2009; Golubitsky et al., 2019). While the use of additional fine-grained spatial structure in the recurrent connectivity with additional pools may generalize our model to this setting, many other forms of visual multistability often involve multiple mutually exclusive three-dimensional interpretations of a single static two-dimensional image due to a lack of depth or orientation cues (Kornmeier and Bach, 2012), for which alternative model settings may be more appropriate in studying their mechanisms.

As multistable perception is documented across sensory systems (Holcombe and Seizova-Cajic, 2008; Hupe et al., 2008; Zhou and Chen, 2009), possibly manifesting from universal organizing principles, computational models have taken diverse creative approaches to granting important mechanistic insights. In the context of audition (Nguyen et al., 2020), for example, one recent study utilized a model featuring two noisy accumulators associated with different ambiguous stimuli, similar to how the dominance metric in this work incorporates firing rate data corresponding to the two monocular stimuli in order to produce the choice of visual percept. However, in the context of auditory bistable perception, the accumulators were forced by data-driven Poisson distributed inputs based on spike count data from the primary auditory cortex, showing that when the saturation strength, akin to adaptation in the present work, is close to the boundary that yields noise-driven attractor dynamics, perceptual alternations agreeing with key experimental observations manifest without the direct use of competition. Another recent study presented a compelling alternative mechanistic framework for binocular rivalry, replacing slow microscopic adaptation with an accumulation process carried out by pools of neurons in the first layer that fed into pools of competing neurons in the second layer (Cao et al., 2021). This alternative framework used out-of-equilibrium dynamics to reproduce common experimental findings and, beyond most existing mechanistic models, further agreed with detailed experimental observations in that it both demonstrated sequential dependence in dominance durations and preserved the statistics of dominance durations even when the contrast of the monocular stimuli was broadly adjusted.

In contrast to binocular rivalry, when the two monocular stimuli are instead sufficiently similar, binocular fusion may occur and result in a constant single image percept, with hysteretic transitions between rivalry and fusion as the similarity of the monocular stimuli varies (Nelson, 1975; Buckthought et al., 2008). How the competition mechanism is counteracted to shift from rivalry to fusion in a unified manner is an important issue (Wilson, 2017) and may be further explored in the two-pool context in this work, for which it is possible to directly manipulate the monocular stimuli and measure the resulting percept given a reconstruction paradigm. While we use a particular monocular stimulus recovery method based on the dynamics of the rivalry metric and compressive sensing techniques applied to the derived input-output mapping given by Equation (2) for the dominant pool, it may also be possible to produce a reasonable percept based on the average of reconstructions generated using the input-output mappings for both the dominant and suppressed pools. Intuitively, for rivalrous dynamics it is likely that the reconstruction from the suppressed pool would only add minimal noise to the reconstruction generated from the dominant pool. In the case of fusion, this averaging approach could coherently generate a single fused percept and potentially yield a more unified understanding of these perceptual phenomena. In a similar vein, the impact of monocular contrasts in fusion for similar images and rivalry for highly distinct stimuli may be analyzed in this context (Wilson, 2017).

There is both theoretical and experimental evidence that the spreading of perceptual waves during alternations in dominance is associated with binocular rivalry (Wilson et al., 2001). In a perceptual transition, a perceptual wave pattern associated with a specific stimulus manifests locally and then spatially spreads with time to eventually eclipse the competing pattern, closely aligning with the propagation speed of traveling wave dynamics of cortical activity in V1 functional magnetic resonance imaging data (Lee et al., 2007). While feedforward connectivity with spatially localized structure akin to receptive fields was examined in Section 2.5, it would be informative to also provide a spatial arrangement in the recurrent connectivity for the two competing neuronal pools, potentially connecting neurons with analogous orientation selectivity, and examine when as well as how this imbues the system with spreading waves of dominance in perceptual alternations for different classes of monocular stimuli.

Giving further credence to empirical evidence that individuals with autism demonstrate weaker binocular rivalry (Robertson et al., 2013; Spiegel et al., 2019), we have systematically demonstrated that the percept alternation rate in binocular rivalry decreases as the network inhibitory connections diminish in strength akin to the reduced impact of GABA hypothesized to be present in the autistic brain (Robertson et al., 2016; Mentch et al., 2019). At the same time, there is still great debate regarding the excitation/inhibition imbalance hypothesis for autism (Happe et al., 2006; Dinstein et al., 2012), and further investigation, both theoretical and experimental, is warranted. Some experimental measurements suggest that the autistic brain is alternatively subject to extraneous excitation (Vattikuti and Chow, 2010) and genetic studies associating excitation or inhibition with autism generally fail to demonstrate a clear directional impact on synaptic transmission (Chao et al., 2010), indicating that continued interplay between experimental advances and mathematical theory is necessary to provide a more parsimonious characterization of the autistic brain.

Though we assumed the activity of the downstream neurons to be prescribed by integrate-and-fire model dynamics, we could similarly investigate whether our framework for rivalrous alternations is robust to more biologically realistic single-neuron models, such as the exponential integrate-and-fire and Hodgkin-Huxley models (Hodgkin and Huxley, 1952; Barranca et al., 2014a). While such models present significant conceptual obstacles in deriving new network input-output mappings crucial to percept reconstructions and may not be analytically tractable, we have developed a methodology for constructing data-driven input-output maps across two layers of excitatory neurons (Barranca et al., 2021) and preliminary work indicates this approach may be extended to balanced networks. Considering that binocular rivalry has provided a unique and fruitful lens through which neuronal computation in the visual system can be understood, linking a dynamic percept to constant external drive, we expect that going forward analogous model-based analysis may help to characterize mechanisms for stimulus encoding and multistability in other sensory systems (Holcombe and Seizova-Cajic, 2008; Hupe et al., 2008; Zhou and Chen, 2009).

## 4. Methods

### 4.1. Network input-output mapping derivation

Under the assumption of irregular neuronal network activity prescribed by Equation (1), in this section we derive a linear input-output relationship crucial to our framework for reconstructing monocular stimulus inputs in Section 2.2. To uncover the underlying network mapping, we estimate the expected voltage for each downstream neuron in the long-time limit. For analytical tractability, we begin by approximating the net effect of all recurrent and feedforward inputs into a given downstream neuron. In considering the recurrent interactions, it is useful to observe that the input into a particular neuron is a spike train summed over the action potentials generated from a relatively large number of neighboring neurons. Since the firing events of neurons in the balanced regime are only weakly correlated, the summed spike train input over a large number of incoming neuronal impulses asymptotically approaches a Poisson point process (Cinlar, 1972). Moreover, because the external input into each pool is constant, we arrive at a simpler statistical characterization of the neuronal inputs. Note that while we derive this input-output mapping for the downstream neurons in the first pool for concreteness, the corresponding mapping for neurons in the second pool is given by interchanging the 1 and 2 subscripts in the equations below.

Since the voltage of each downstream neuron is reset to *V*^*Re*^ upon firing, we consider Equation (1) with initial condition ${v_{1}}_{k}^{i}\left(t=0\right)=V^{Re}$ for *k* = *E, I*. Assuming Poisson spike train inputs as described above, the solution to the corresponding initial value problem gives the subthreshold membrane potential trajectory for the *i*^th^ neuron of the *k*^th^ population in the first pool and may be expressed as


v1ki(t)=VRe+∑j=1j≠iNER1kEijΨ1Ej(t)+∑j=1j≠iNIR1kIijΨ1Ij(t)

(5a) +∑j=1j≠iNEC2kEijΨ2Ej(t)+(1-e-gLtgL)∑jF1kijp1j

(5b) $$\begin{array}{l} {{\text{Ψ}}_{z}}_{l}^{j}\left(t\right)=\overset{{T_{z}}_{l}^{j}\left(t\right)}{\underset{s=1}{\sum }}e^{-g_{L}\left(t-{U_{z}}_{l,s}^{j}\left(t\right)\right)}, \end{array}$$

where ${R_{1}}_{kl}^{ij}\cdot {{\text{Ψ}}_{1}}_{l}^{j}\left(t\right)$ yields the total recurrent spike train input from the *j*^th^ neuron of the *l*^th^ population in the first pool into the *i*^th^ neuron of the *k*^th^ population in the first pool at time *t* and ${T_{z}}_{l}^{j}\left(t\right)$ denotes the total number of spikes transmitted by the *j*^th^ neuron of the *l*^th^ population in the *z*^th^ pool through time *t*. Note that here ${T_{z}}_{l}^{j}\left(t\right)$ is described approximately by a Poisson distribution with average number of events ${m_{z}}_{l}^{j}t$, where ${m_{z}}_{l}^{j}$ is the long-time average firing rate of the *j*^th^ neuron of the *l*^th^ population in the *z*^th^ pool.

Corresponding to ${T_{z}}_{l}^{j}\left(t\right)$, the spike train input from the *j*^th^ neuron of the *l*^th^ population in the *z*^th^ pool has spike times denoted by ${U_{z}}_{l,s}^{j}\left(t\right)$, for *s* = 1, 2, … , which we assume are uniformly distributed in the time interval [0, *t*] based on the irregularity of firing events. Hence, the term in Equation (5) given by ${Y_{z}}_{l,s}^{j}\left(t\right)=e^{-g_{L}\left(t-{U_{z}}_{l,s}^{j}\left(t\right)\right)}$ is a random variable that takes on values in the interval $\left[e^{-g_{L}t},1\right]$. Since the probability density function for random variable ${U_{z}}_{l,s}^{j}\left(t\right)$ is $P_{{U_{z}}_{l,s}^{j}\left(t\right)}\left(u\right)=1/t$ in its support, it follows that random variable ${Y_{z}}_{l,s}^{j}\left(t\right)$ has probability density function $P_{{Y_{z}}_{l,s}^{j}\left(t\right)}\left(y\right)=1/\left(g_{L}ty\right)$ for $y\in \left[e^{-g_{L}t},1\right]$ and expected value $\left(1-e^{-g_{L}t}\right)/\left(g_{L}t\right)$.

Since ${{\text{Ψ}}_{z}}_{l}^{j}\left(t\right)$ is a sum of nearly independent identically distributed random variables, it has expected value $\frac{1-e^{-g_{L}t}}{g_{L}t}{m_{z}}_{l}^{j}t$ for time horizon *t*. Temporarily disregarding the impact of the reset condition, the expected voltage of the *i*^th^ neuron of the *k*^th^ population in the first pool at time *t* is thus approximately


$$\begin{array}{l} {\tilde{v_{1}}}_{k}^{i}(t)=V^{Re}+\frac{1-e^{-g_{L}t}}{g_{L}}\cdot (\sum_{j}F_{1}{}_{k}^{ij}p_{1}{}^{j}\\ \;+\sum_{\begin{array}{c} j=1\\ j\neq i \end{array}}^{N_{E}}R_{1}{}_{kE}^{ij}m_{1}{}_{E}^{j}+\sum_{\begin{array}{c} j=1\\ j\neq i \end{array}}^{N_{I}}R_{1}{}_{kI}^{ij}m_{1}{}_{I}^{j}+\sum_{\begin{array}{c} j=1\\ j\neq i \end{array}}^{N_{E}}C_{2}{}_{kE}^{ij}m_{2}{}_{E}^{j}). \end{array}$$

As *t* → ∞, we have that ${\tilde{v_{1}}}_{k}^{i}\left(t\right)$ approaches


$$\begin{array}{l} {\tilde{v_{1}}}_{k}^{i}=V^{Re}+\frac{1}{g_{L}}\cdot (\sum_{j}F_{1}{}_{k}^{ij}p_{1}{}^{j}\\ \;+\sum_{\begin{array}{c} j=1\\ j\neq i \end{array}}^{N_{E}}R_{1}{}_{kE}^{ij}m_{1}{}_{E}^{j}+\sum_{\begin{array}{c} j=1\\ j\neq i \end{array}}^{N_{I}}R_{1}{}_{kI}^{ij}m_{1}{}_{I}^{j}+\sum_{\begin{array}{c} j=1\\ j\neq i \end{array}}^{N_{E}}C_{2}{}_{kE}^{ij}m_{2}{}_{E}^{j}). \end{array}$$

The true expected voltage, ${\bar{v_{1}}}_{k}^{i}$, will be lower than ${\tilde{v_{1}}}_{k}^{i}$ since upon reaching the dynamic threshold ${\theta_{1}}_{k}^{i}$, the voltage is instantaneously reset down to *V*^*Re*^. As a result of this non-linearity, since the change in voltage due to a given action potential is $-\left({\theta_{1}}_{k}^{i}-V^{Re}\right)$ occurring with rate ${m_{1}}_{k}^{i}$, it follows that the true long-time expected voltage for the *i*^th^ neuron in the *k*^th^ population of the first pool, ${\bar{v_{1}}}_{k}^{i}$, is approximately


(6) $$\begin{array}{ll} {\bar{v_{1}}}_{k}^{i}= & V^{Re}+\frac{1}{g_{L}}\cdot (\sum_{j}F_{1}{}_{k}^{ij}p_{1}{}^{j}\;\\ & +\sum_{\begin{array}{c} j=1\\ j\neq i \end{array}}^{N_{E}}R_{1}{}_{kE}^{ij}m_{1}{}_{E}^{j}+\sum_{\begin{array}{c} j=1\\ j\neq i \end{array}}^{N_{I}}R_{1}{}_{kI}^{ij}m_{1}{}_{I}^{j}\\ & +\sum_{\begin{array}{c} j=1\\ j\neq i \end{array}}^{N_{E}}C_{2}{}_{kE}^{ij}m_{2}{}_{E}^{j}-m_{1}{}_{k}^{i}({\bar{\theta_{1}}}_{k}^{i}-V^{Re})), \end{array}$$

where ${\bar{\theta_{1}}}_{k}^{i}$ is the time-averaged firing threshold for the *i*^th^ neuron in the *k*^th^ population of the first pool. Linking the evoked downstream network dynamics to the monocular image input given by **p_1_**, Equation (6) is the linear input-output mapping that we will leverage to estimate the encoded percepts in Section 2.2 and is expressed in matrix form in Equation (2).

With this mapping in hand, we next derive conditions on the model parameters that theoretically produce balanced dynamics in the dominant pool. The excitatory neurons in the suppressed pool are sufficiently quiescent such that the cross-pool inputs in Equation (6) determined by matrix **C_2_** have relatively little impact on the expected voltage offset from *V*^*Re*^, and thus we derive balance conditions for the dominant pool ignoring the competitive long-range inputs included in our full model. Viewing the dynamics at the population level, since each neuron in the *l*^th^ population of the first pool is expected to fire at rate given by the long-time population-averaged firing rate _*m*_1_*l*_ and each neuron in the *k*^th^ population of the first pool is expected to receive *K* incoming recurrent connections from the *l*^th^ population of the first pool with individual connection strength $R_{kl}/\sqrt{K}$, the expected total recurrent input from the *l*^th^ population in the first pool into a neuron in the *k*^th^ population of the first pool is approximately $R_{kl}{m_{1}}_{l}\sqrt{K}$. Similarly, we assume the expected feedforward input into a downstream neuron in the *k*^th^ population of an arbitrary pool is of magnitude $\sqrt{K}f_{k}m_{0}$, with O(1) parameters *m*_0_ and *f*_*k*_ respectively scaling the overall and relative feedforward drive strengths for the excitatory and inhibitory populations. As a result, taking the expectation of Equation (6) over all network realizations, we approximate the expected offset in voltage from *V*^*Re*^ due to both recurrent and feedforward inputs for a downstream neuron in the *k*^th^ population of the first pool as


(7) $$\begin{array}{l} {d_{1}}_{k}=\sqrt{K}\frac{1}{g_{L}}\left(f_{k}m_{0}+R_{kE}{m_{1}}_{E}+R_{kI}{m_{1}}_{I}\right). \end{array}$$

Seeking irregular activity indicative of balance within the dominant pool, the expected voltage for a given downstream neuron must remain finite as *K* → ∞ in the large-network limit. Based on Equation (7), which neglects the weak cross-pool drive and assumes statistically equivalent constant feedforward drive in the two pools for fairness in rivalry, it must be that *f*_*k*_*m*_0_+*R*_*kE*__*m*_1_*E*_+*R*_*kI*__*m*_1_*I*_ is $O(\sqrt{K})$ and vanishes as *K* → ∞ (van Vreeswijk and Sompolinsky, 1998). The resultant constraints for both the excitatory and inhibitory populations composing the first pool provide a system of two linear equations with solution


(8a) $$\begin{array}{l} {m_{1}}_{E}=\frac{|R_{II}|f_{E}-|R_{EI}|f_{I}}{R_{IE}|R_{EI}|-R_{EE}|R_{II}|}m_{0} \end{array}$$

(8b) $$\begin{array}{l} {m_{1}}_{I}=\frac{R_{IE}f_{E}-R_{EE}f_{I}}{R_{IE}|R_{EI}|-R_{EE}|R_{II}|}m_{0}, \end{array}$$

indicating a linear scaling of the population-averaged firing rates with the overall feedforward input strength parameter *m*_0_.

Requiring that both the excitatory and inhibitory population-averaged firing rates are non-negative and finite in the balanced dynamical regime, Equation (8) provides parameter bounds referred to as balance conditions,


(9) $$\begin{array}{l} \frac{f_{E}}{f_{I}}>\frac{\left|R_{EI}\right|}{\left|R_{II}\right|}>\frac{R_{EE}}{R_{IE}}, \end{array}$$

which are theoretically necessary for balanced dynamics in the dominant pool in the large-network limit. The linear scaling of downstream neuronal firing rates with feedforward input strength demonstrated by Equation (8) holds theoretically on the population level when the balance conditions are satisfied and in Figure 1E we empirically examine the population-averaged firing rates for the model network in response to increasingly large feedforward inputs by increasing the overall feedforward scaling strength *m*_0_ across a family of simulations. We observe that as the mean feedforward drive is increased, the average firing rate of both the excitatory and inhibitory populations in a given pool linearly increases over a wide range of scalings, agreeing with the estimate computed in the large-network limit and corroborating the accuracy of the derived input-output mapping.

While the original balanced network theory was derived in the context of constant and homogeneous excitatory external inputs driving the two populations in a single pool of binary neurons (van Vreeswijk and Sompolinsky, 1996), it is important to emphasize that in our two-layer I&F network model the excitatory external input vectors are instead heterogeneous and determined by the pixels composing the monocular image stimuli. Nonetheless, as long as the stimulus strength scalings for the excitatory and inhibitory populations in a given pool are such that *f*_*E*_>*f*_*I*_ for these heterogeneous external inputs, the classical balance conditions given by Equation (9) hold on average and balanced dynamics are primarily preserved in the dominant pool.

### 4.2. Compressive sensing theory

Compressive sensing theory shows that for sparse data, the amount of measurements necessary for a successful reconstruction is determined by the number of dominant non-zero components in the data (Candes et al., 2006; Donoho, 2006). This suggests that optimally reconstructing sparse data from relatively few samples requires selecting the sparsest viable reconstruction that agrees with the collected information, since the resultant signal is most compressible. By leveraging the sparse structure of signals, CS offers a significant improvement in efficiency from the classical Shannon-Nyquist theorem, which asserts that the sampling rate should be determined by the full bandwidth of the data (Shannon, 1949). Considering that common signals and sensory stimuli, such as scenes, soundwaves, and odorants, are each sparse in a certain domain (Field, 1994; Markram et al., 1997), CS theory has amassed numerous and broad scientific applications (Lustig et al., 2007; Dai et al., 2009; Berger et al., 2010; Gross et al., 2010).

The reconstruction of time-invariant data from a small number of samples generates a highly underdetermined linear system. For an *n*-component signal, **x**, a total of *m* discrete samples of **x** can be represented by **Ax**, where **A** is an *m*×*n* measurement matrix composed of rows that each prescribe a set of measurement weights. This yields an *m*-component measured signal, **b**, where *m*≪*n*. Assuming that signal **x** is sufficiently sparse, CS theory shows for a large class of measurement matrices that minimizing $|x{|}_{\ell_{1}}=\sum_{i=1}^{n}\left|x_{i}\right|$ yields the sparsest reconstruction consistent with the measurements (Candes and Wakin, 2008; Bruckstein et al., 2009). The resultant optimization problem


(10) $$\arg\underset{{}_{x\in R^{n}}}{\min}|x{|}_{\ell_{1}}\text{ subject to }Ax=b$$

is well-researched and can be efficiently solved using numerous fast numerical methods, thus yielding a tractable reconstruction methodology for high-dimensional data with a sparse representation (Tropp and Gilbert, 2007; Donoho and Tsaig, 2008).

The recovered signal exhibits variations depending on the optimization algorithm used, but the minimal relative reconstruction errors generated across methods are quite comparable for our work. Moreover, if signal **x** is not sparse in the sampled domain and is instead sparse under a transform, *L*, then the linear system $\phi \hat{x}=b,$ where **ϕ** = **A***L*^−1^ and $\hat{x}=Lx$, can be considered similarly. In the case of the natural scenes analyzed in our model, the monocular image input is indeed non-sparse in the original pixel domain but is instead sparse in a frequency-based domain. For concreteness, we utilize the two-dimensional discrete-cosine transform to take advantage of the sparse stimulus structure underlying our percept reconstructions (Heil and Walnut, 1989; Barranca et al., 2014b).

In determining an appropriate sampling scheme, it is important to note that measurement matrices exhibiting little correlation among their columns and well-preserving signal magnitudes, such as those containing independent identically distributed elements, are typically feasible candidates (Baraniuk, 2007; Candes and Wakin, 2008). While diverse matrices demonstrating sufficient randomness in their structure have been proven to suitably exhibit these properties (Candes et al., 2006; Candes and Wakin, 2008), there are other sampling matrices with more complex structure that are highly successful in CS signal reconstructions but not analytically tractable. Improved sampling matrices may be generated, for example, from experimental insights or machine learning techniques (Barranca, 2021). As a result, numerous sampling schemes are amenable to CS recovery and they may be adapted based on logistical constraints or a priori knowledge of signal characteristics.

## Data availability statement

The original contributions presented in the study are included in the article/supplementary material, further inquiries can be directed to the corresponding author.

## Author contributions

All authors listed have made a substantial, direct, and intellectual contribution to the work and approved it for publication.
